# Evaluation of stripe rust resistance and analysis of resistance genes in wheat genotypes from Pakistan and Southwest China

**DOI:** 10.3389/fpls.2024.1494566

**Published:** 2024-12-09

**Authors:** Sakina Abbas, Yunfang Li, Jing Lu, Jianming Hu, Xinnuo Zhang, Xue Lv, Armghan Shahzad, Donghui Ao, Maryam Abbas, Yu Wu, Lei Zhang, Muhammad Fayyaz

**Affiliations:** ^1^ Chengdu Institute of Biology, Chinese Academy of Sciences, Chengdu, China; ^2^ University of Chinese Academy of Sciences, Beijing, China; ^3^ National Institute of Genomics and Advance Biotechnology, Pakistan Agriculture Research Council, Islamabad, Pakistan; ^4^ Department of Computer Science, Iqra University, Islamabad, Pakistan; ^5^ Crop Diseases Research Institute, Pakistan Agriculture Research Council, Islamabad, Pakistan

**Keywords:** stripe rust, gene pyramiding, marker-assisted selection, *Yr* genes, molecular markers

## Abstract

**Introduction:**

Stripe rust, caused by *Puccinia striiformis* f. sp. *tritici*, poses a significant threat to wheat quality and production worldwide. The rapid evolution of *Pst* races caused several resistance genes to be ineffective.

**Methods:**

This study evaluated stripe rust resistance genes in 349 Pakistan and Southwest China genotypes. We utilized previously published functional and linked molecular markers to detect 13 major stripe rust resistance genes: *Yr5, Yr9, Yr10, Yr15, Yr17, Yr18, Yr26, Yr29, Yr30, Yr36, Yr48, Yr65*, and *YrSp*. Field evaluations assessed IT and resistance levels, while the impact of gene combinations on resistance was also analyzed.

**Results:**

Field evaluations showed that over 60% of Chuanyu wheat, 50% of recent Pakistani cultivars, and 20% of historic Pakistani lines were resistant to current stripe rust races. In Chuanyu wheat, the dominant genes were *Yr17, YrSp*, and *Yr48*; however, *Yr17, Yr26*, and *YrSp* were overused, while *Yr36* was absent, and *Yr18* was rare. In historic lines, *Yr5, Yr17, Yr18*, and *Yr26* were prevalent, with *Yr15, Yr26*, and *YrSp* demonstrating effective resistance against current stripe rust races. Furthermore, the study identified specific combinations of *Yr* genes (*Yr26+Yr48, Yr29+Yr5, Yr26+Yr30*, and *Yr30+Yr17*) that enhanced resistance to *Pst*.

**Discussion:**

This research highlights effective resistance genes and gene combinations for stripe rust in wheat and emphasizes the deployment of durable resistance. The findings guide the strategic use of these genes in breeding programs aimed at developing durable resistance in wheat genotypes in Pakistan and Southwest China.

## Introduction

Wheat (*Triticum aestivum.* L.) is one of the world’s most important staple crops, serving as a primary food source for a large percentage of the global population (Kiani et al., 2021) and providing nearly 18% of the calories and 19% of the protein for human consumption (Erenstein et al., 2022). There will be a substantial increase in the demand for wheat as the world’s population grows. Despite such demand, wheat production is challenged by several potential constraints, among which biotic stresses are exceptionally severe (Figueroa et al., 2017). Among others, stripe rust, induced by *Puccinia striiformis* f. sp. *Tritici* (*Pst*), is believed to be the most devastating and has been frequently reported to affect the production of wheat dramatically. This disease can cause yield reduction ranging from 10% to 70% on susceptible cultivars, with possible losses reaching 100%, particularly during intense epidemic time (Chen, 2020; Kokhmetova et al., 2021). Owing to the genetic diversity and rapid adaptation of virulent *Pst* races, the disease annually affects regions where climatic conditions and cropping practices are favorable for stripe rust (Chen, 2005). Ground data suggested that it become widespread in 41 primary wheat-producing regions worldwide, including China and Pakistan (Ma et al., 2023).

The surveillance for stripe rust in China has shown that the southwest and northwest regions are more vulnerable to the pathogen throughout the summer (Zhao and Kang, 2023). Both regions serve as significant origins and hubs for the *Pst* pathogen, contributing to its spread (Xu et al., 2019; Zhao and Kang, 2023). In China, there has been a substantial yield reduction caused by stripe rust outbreaks in various years, resulting in losses of 3.2 million metric tons (Mmt) in 1964, 1.8 Mmt in 1990, 1.3 Mmt in 2002, and 1.5 Mmt in 2017 (Wan et al., 2007; Chen et al., 2023). In Pakistan, areas like the northern and central-western regions are more prone to stripe rust (Bahri et al., 2011; Habib et al., 2020; Mehmood et al., 2020). Previous reports suggested that approximately 5.8 million hectares, comprising 70% of the wheat-growing area, is susceptible to stripe rust (Kiani et al., 2021), which accounts for around 70%–75% of Pakistan’s wheat cultivation area (Sobia et al., 2010; Habib et al., 2020). To address the ongoing rapid development of stripe rust, especially in areas where outbreaks are common, such as China and Pakistan, the most economical and sustainable method is to develop resistant cultivars (Kalogiannidis et al., 2022; Rebouh et al., 2023).

To control stripe rust genetically, there are currently more than 83 known *Yr* resistance genes that are spread throughout 21 chromosomes (Maccaferri et al., 2015; Mcintosh et al., 2016). Only eight *Yr* genes*—Yr36* (Fu et al., 2009), *Yr18* (*Lr34*) (Lagudah et al., 2009), *Yr46* (*Lr67*) (Hiebert et al., 2015), *Yr15* (Klymiuk et al., 2018), *Yr5/YrSp* (Marchal et al., 2018), *Yr79*, *YrAS2388*, and *YrU111*—have been functionally validated, while *Yr10* was being provisionally cloned (Ni et al., 2023). The *Yr5* gene, derived from *T*. *spelta Album*, is responsible for conferring resistance to *Pst* (Macer, 1966). Nevertheless, it is noteworthy that only a limited number of genotypes capable of overcoming *Yr5* resistance have been identified so far, specifically in Australia (Mclntosh et al., 1995), India (Prashar et al., 2015), Turkey (Tekin et al., 2021), and China (Liu et al., 2020). The *Yr10* resistance gene is effective in providing resistance against stripe rust in Pakistan (Sobia et al., 2010), Iran (Sarfaraz et al., 2013), India (Rani et al., 2019), and China (Huang et al., 2020). The primary gene responsible for resistance to stripe rust, *Yr15*, was discovered in *Triticum dicoccoides* accession G-25 and is located on chromosome 1BS (Klymiuk et al., 2021). Breeders across the globe have made use of the *Yr17* gene, which confers resistance to stripe rust in wheat (Helguera et al., 2003). The *Yr18* genes have been employed in breeding efforts for a century, and thus far, no evidence of pathogen adaptation has been discovered (Lagudah et al., 2009).

Recent advancements in molecular technologies have revolutionized plant breeding through the integration of molecular markers to improve breeding precision and efficiency (Sun et al., 2024). In contrast to conventional approaches that emphasize phenotypic selection, the use of molecular markers allows for the precise identification and monitoring of specific genes and quantitative trait loci (QTLs), thereby enhancing the processes of marker-assisted and genomic selection (Hasan et al., 2021; Lema, 2018). The advancement of these tools has significantly enhanced the development of better crop varieties by offering thoughtful insights into genetic diversity and complex traits, thereby facilitating more precise and effective breeding strategies (Nadeem et al., 2017). Significant advancements have been achieved in the development of stripe rust-resistant cultivars through breeding in both countries. However, the breeding program has been significantly impeded by the rapid evolution of pathogen races, which remain a global hazard to wheat cultivation (Bhavani et al., 2021). The objectives of this study are to (1) evaluate stripe rust resistance in Pakistani and Chuanyu wheat genotypes, (2) illustrate the distribution of major *Yr* genes by using molecular markers in Pakistani and Chuanyu wheat genotypes, and (3) analyze the effectiveness of major *Yr* genes and their combinations. This study provides valuable information for an efficient *Yr* gene utilization strategy to improve *Pst* resistance in Pakistani and Chinese wheat genotypes.

## Materials and methods

### Plant material

**Figure 1:**
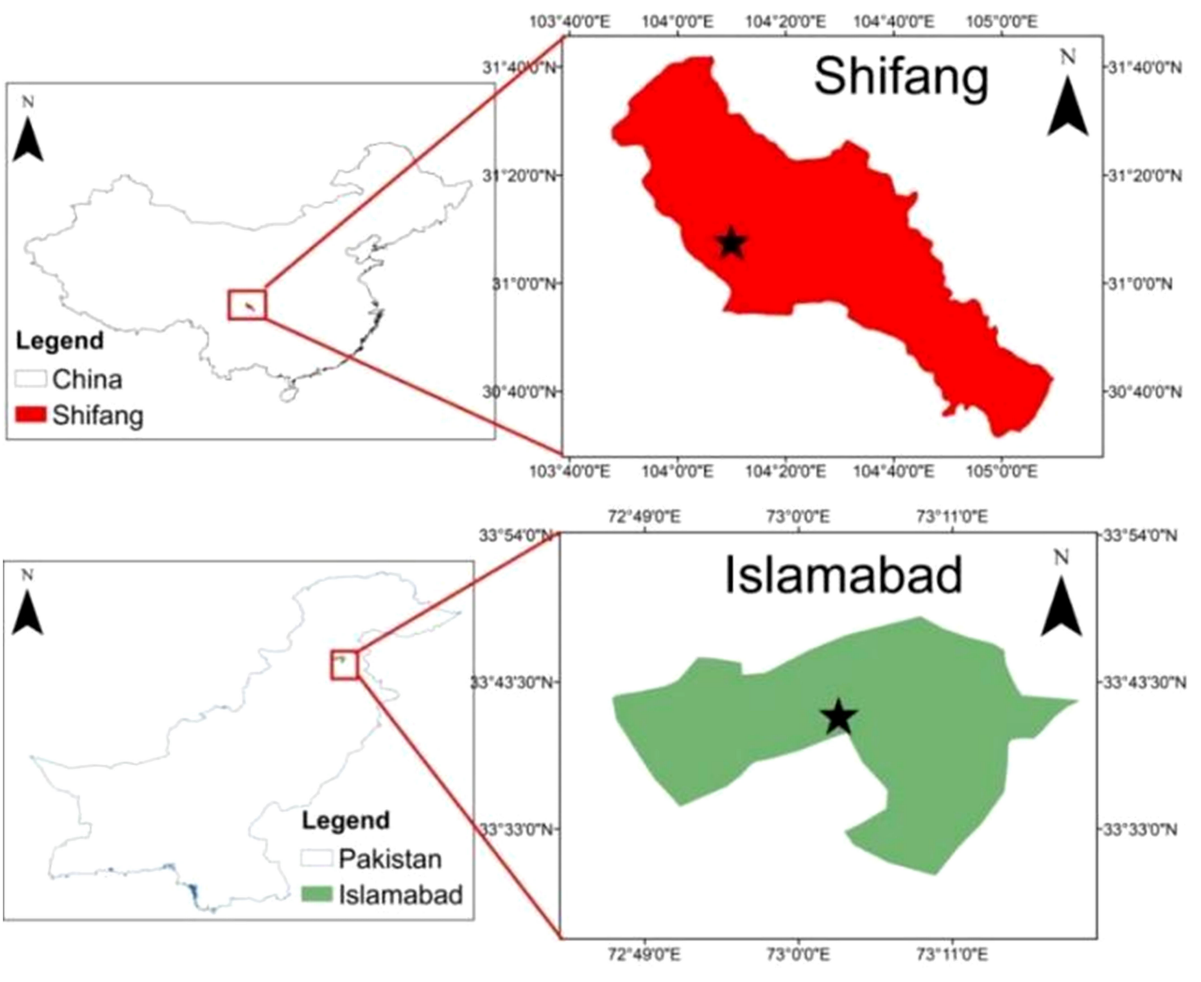
A map of the study area effectively demarcates the geographical boundaries and the experimental sites of the research area.

A panel of 349 genotypes was evaluated in this study and divided into three groups. The first group comprises 222 Chuanyu wheat genotypes (“Chuan” means Sichuan and “Yu” means breeding or cultivation), which represent germplasm from Southwest China (including both varieties and advanced lines; out of 222, only 16 were varieties). The second group represents the 80 historic lines of Pakistan, and the third group includes 47 recent cultivars of Pakistan (detailed information about the germplasm is available in **Supplementary File S1** (**Supplementary Tables S6**-**S8**). The first experimental field trial was conducted at Shifang (104′11 E, 31.6 N, elevation 521) (**Figure 1**), Sichuan Province, during November 2022–2023. Plant material for the molecular test was collected in January 2023, and field evaluation for *Pst* was performed in April–May 2023. Each experimental plot was designed as a random block, and a single seed genotype was sown in a single row, and general agronomic practices were performed until harvest.

The Pakistani wheat genotype field experiment was carried out at the National Agriculture Research Center, Islamabad (33.6667° E, 073.1667° N, elevation 516.11 (**Figure 1**). In wheat growing year November 2021–2022 and November 2022–2023, field evaluations of stripe rust were carried out in both years. Each variety was sown in a single row by maintaining a 30-cm row-to-row distance. A single line of susceptible cultivar Morocco and some local checks were sown repeatedly after every 10 lines as checks to spread rust infection. The inoculum was sprayed via a ULV sprayer onto the rust-resistant cultivar Morocco during the booting stage in both years and at both field sites. This particular stage was chosen because it aligns with the optimal weather conditions for rust spread. Afterward, the percentage of rust infection and severity was recorded once the Morocco genotype reached a severity level of 70%–80%.

### Pathogen inoculation

**Figure 2:**
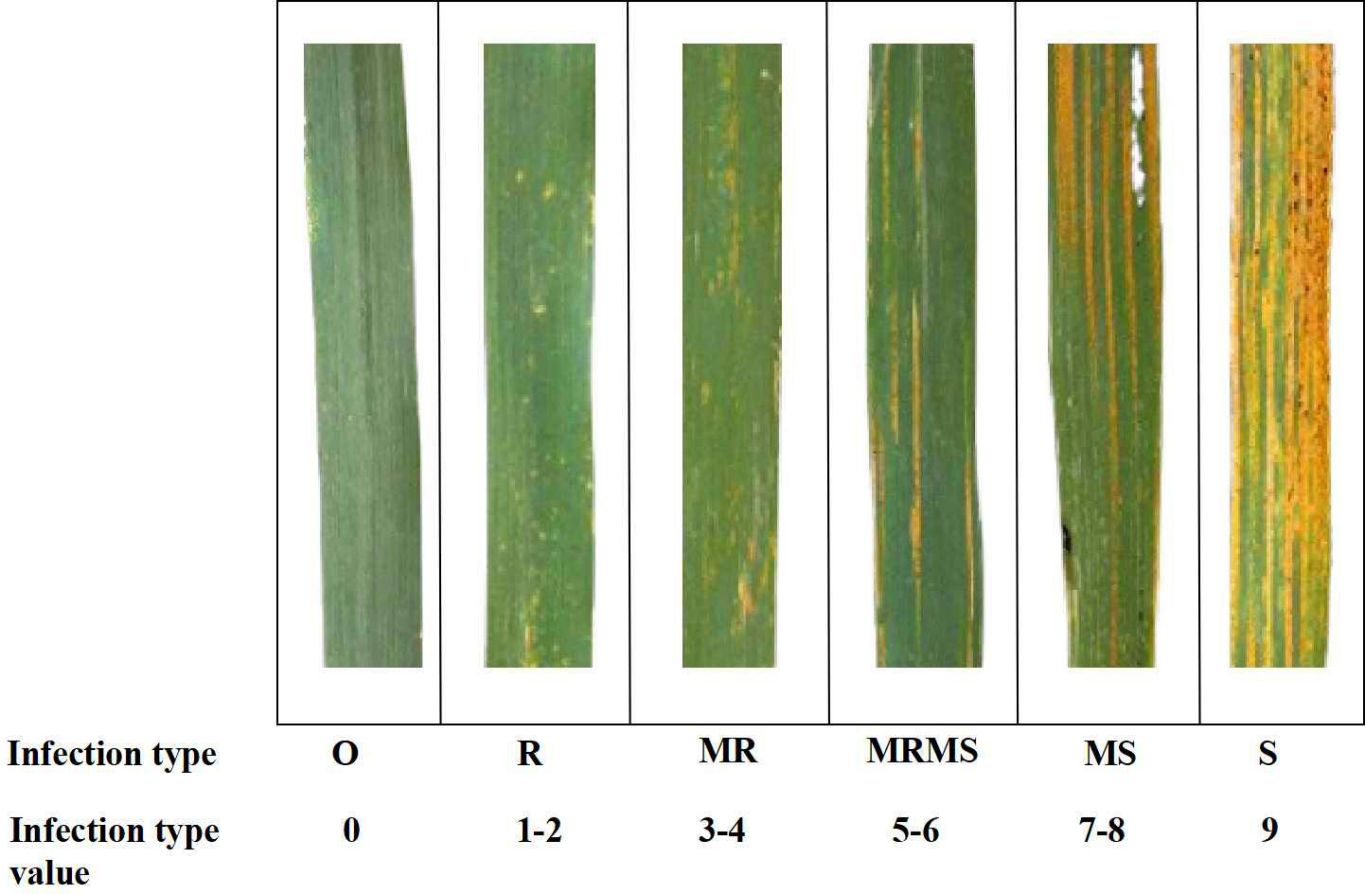
Phenotypic evaluation of genotypes with reference to the progression of stripe rust infection in wheat genotypes (O, no visible infection; R, resistant; MR, moderately resistant; MS, moderately susceptible; S, susceptible).

The stripe rust infection type (IT) value was evaluated using a standard 0–9 identification system (**Figure 2**) according to the modified Cobb’s scale used by Mahmoud et al. (2015).

For artificial inoculation in Chuanyu genotypes, mixed races of CYR32, CYR33, Hybrid46, and Guinong22 were used, whereas for Pakistani genotypes mixed races of Pstv-37, PK07-4, PK07-12, and PK08-2 used to spread infection. Stripe rust IT has been observed three times throughout the adult stage 20 to 23 weeks after sowing.

### DNA extraction and molecular marker genotyping

Approximately 100 mg of fresh leaf tissue was harvested from each seedling, and genomic DNA extraction was carried out by using the CTAB (hexadecyltrimethylammonium bromide) method previously reported by Jilin (2013). The extracted DNA samples were then stored at -20°C for preservation. Following the extraction procedure, the concentration and purity of the DNA samples were accurately measured through agarose gel electrophoresis and using a nanodrop spectrophotometer, respectively, to ensure their quality and suitability for subsequent molecular analyses. PCR was performed to investigate the genetic polymorphism among genotypes that are already known for *Yr* genes. The PCR products were subsequently separated and visualized using 1.5% agarose gel electrophoresis (Sigmon and Larcom, 1996) and 8% non-denaturing polyacrylamide gel electrophoresis, with subsequent staining using silver nitrate (Chevallet et al., 2006). **Supplementary File S1** (**Supplementary Table S4**) presents a comprehensive overview of 24 pairs of functional and linked markers used for the detection of 13 major stripe rust resistance genes (*Yr5*, *Yr9*, *Yr10*, *Yr15*, *Yr17*, *Yr18*, *Yr26*, *Yr29*, *Yr30*, *Yr36*, *Yr48*, *Yr65*, and *YrSp*), some of which are all-stage resistance (ASR). Others are adult plant resistance (APR). The sequences of the markers were downloaded from maswheat.ucdavis.edu/and the Grain Gene website wheat.pw.usda.gov/GG3/.

### Statistical analysis

All the phenotypic and genotypic data were recorded in Microsoft Office Excel 2016. SPSS 20.0 was used to perform a statistical analysis of variance (ANOVA). The Pearson correlation analysis was performed using the online tool SRPlot (www.bioinformatics.com.cn/). Gen stat 22nd Edition and Origin 2024 were used to make graphs.Results

### Field evaluation of *Pst* resistance

**Figure 3:**
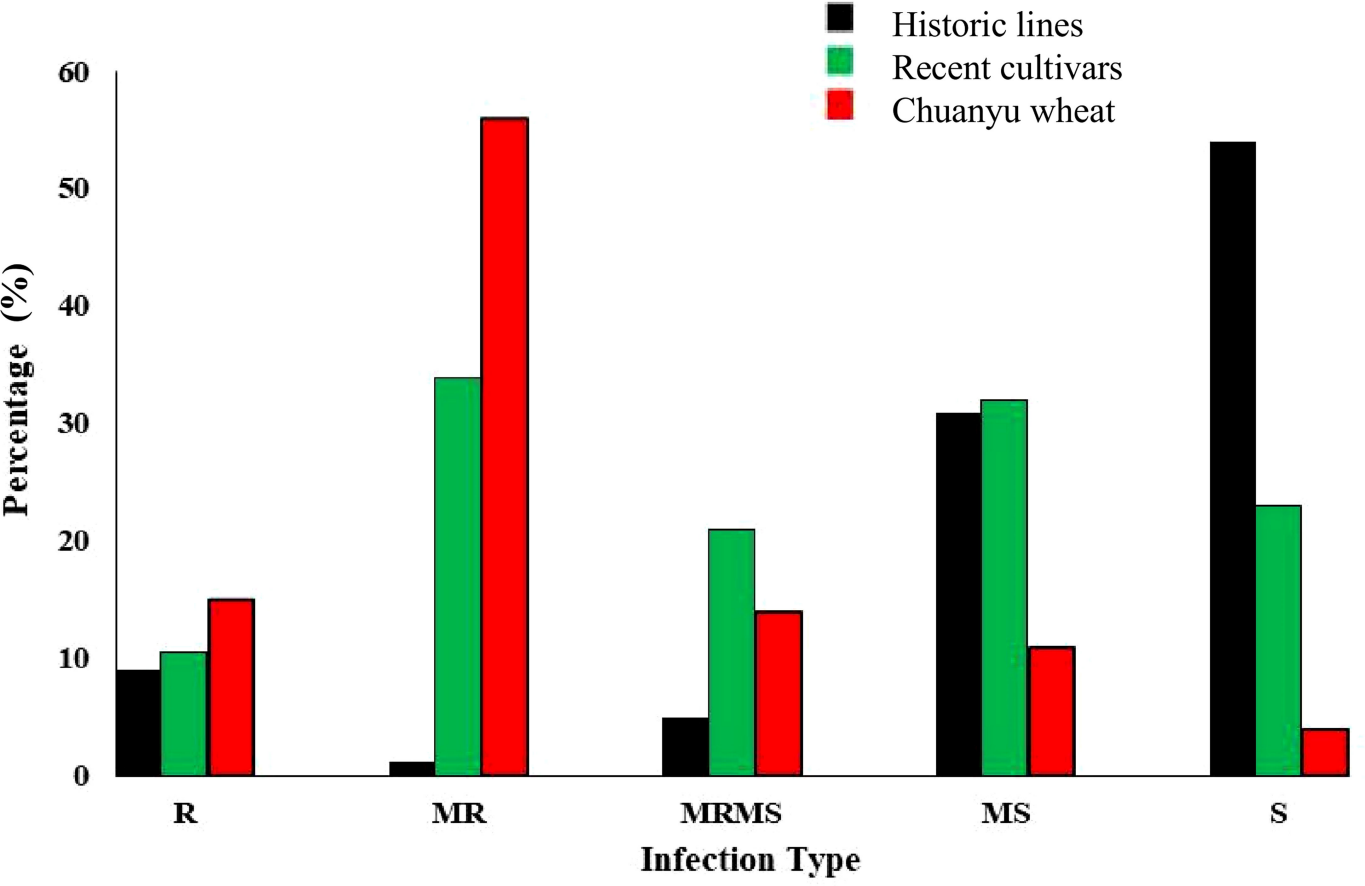
The distribution of infection type on the X-axis in a panel of 349 wheat genotypes are divided into three groups: Chuanyu wheat, recent cultivars, and historical lines of Pakistan, whereas the percentage (%) of genotypes is shown on the Y-axis.

A panel of 349 genotypes at the adult plant stage was evaluated for stripe rust resistance in the field. These entries were grouped into three categories, including historic lines of Pakistan, recent cultivars of Pakistan, and Chuanyu wheat. The analysis of stripe rust in different cultivars indicated varying levels of resistance and susceptibility, as illustrated in **Figure 3**. According to the field response, 9% of historical lines, 10.6% of recent cultivars, and 15% of Chuanyu wheat were resistant, and those with moderate resistance included 1.25% of historical lines, 34% of recent cultivars, and 56% of Chuanyu wheat. The moderate susceptible reaction was shown by 31%, 32%, and 11% of historical lines, recent cultivars, and Chuanyu wheat, respectively. In contrast, 54% of historical lines, 23% of recent cultivars, and only 4% of Chuanyu wheat were found to be susceptible. Remarkably, Chuanyu wheat shows a higher resistance characterized by a higher percentage of resistance and significantly lower susceptibility, while Pakistan’s recent cultivars have moderate resistance, whereas most of Pakistan’s historic lines have lost their resistance against the current stripe rust races in the field (**Tables 1** , **2**).

**Table 1:** Field evaluation of Chuanyu wheat for *Pst* infection.

Infection type	Genotypes
Resistant	R5, R8, R9, R11, R12, R16, R18, R19, R22, R23, R24, R25, R26, R28, R29, R32, R33, R34, R36, R37, R39, R40, R41, R42, R50, R52, R53, R56, R57, R59, R60, R65, R66, R69, R70, R71, R73, R77, R78, R79, R82, R85, R86, R89, R96, R98, R103, R105, R108, R109, R115, R119, R121, R125, R129, R130, R131, R132, R133, R134, R135, R136, R138, R140, R144, R148, R149, R150, R153, R154, R160, R161, R162, R163, R164, R170, R171, R175, R176, R177, R178, R179, R180, R184, R191, R193, R195, R202, R204, R209, R210, R211, R212, R213, R214, R215, R216, R217, R218, R219
Moderately resistant	R1, R3, R7, R10, R13, R14, R15,R17, R20, R21, R30, R31, R35, R38, R43, R44, R45, R46, R47, R51, R58, R61, R63, R64, R67, R68, R72, R74, R75, R76, R83, R84, R87, R88, R93, R94, R95, R97, R102, R104, R106, R110, R111, R113, R114, R117, R118, R120, R122, R123, R124, R126, R127, R128, R137, R139, R143, R145, R147, R151, R155, R165, R166, R167, R168, R169, R172, R173, R174, R181, R185, R187, R188, R189, R190, R192, R194, R196, R197, R198, R199, R201, R203, R205, R207
Moderately susceptible	R6, R27, R48, R49, R54, R55, R62, R80, R81, R90, R91, R92, R99,R100, R101, R107, R112, R116, R142, R152, R183, R186
Susceptible	R2, R4, R141, R146, R156, R157, R158, R159, R200, R206, R208, R220, R222

Genotypes are represented by R1, R2, R3…R222. Resistant, Moderately resistant, moderate susceptible, and susceptible are distinct categories that indicate varying levels of disease resistance, ranging from strong resistance to high susceptibility.

**Table 2:** Field evaluation of historic lines and recent cultivars of Pakistan, which showed different levels of IT (resistant, moderately resistant, moderately susceptible, and susceptible, indicating varying levels of disease resistance, ranging from strong resistance to high susceptibility).

Infection type	Genotypes
Resistant	D1, D24, D37, D42, D45, D47, G1, G3, G5, G6, G7, G8, G21, G40
Moderately resistant	D4, D7, D23, D28, D30, D37, D39, D40, D43, D44, D46, G2, G28, G53, G65, G78
Moderately susceptible	D5, D8, D12, D13, D14, D16, D20, D21, D22, D25, D27, D28, D29, D31, D33, D35, D41, D2, D3, D6, D9, D10, D11, D15, D17, D19, D26, D32, G4,G12, G15, G18, G26, G27, G31, G35, G36, G38, G39, G41, G44, G48, G49, G50, G55, G56, G58, G64, G66, G73, G74, G75
Susceptible	D18, D34, D36, G9, G10, G11, G13, G14, G16, G17, G19, G20, G22, G23, G24, G25, G29, G30, G32, G33,G34, G37, G42, G43, G45, G46, G47, G51, G52, G54, G57, G59, G60, G61, G62, G63, G67, G68, G69, G70, G71, G72, G76, G77, G79, G80

Historic lines genotypes are represented by G1, G2, G3…G80, whereas recent cultivars are represented by D1, D2, D3…D47.

### Frequency distribution of *Yr* genes

**Figure 4:**
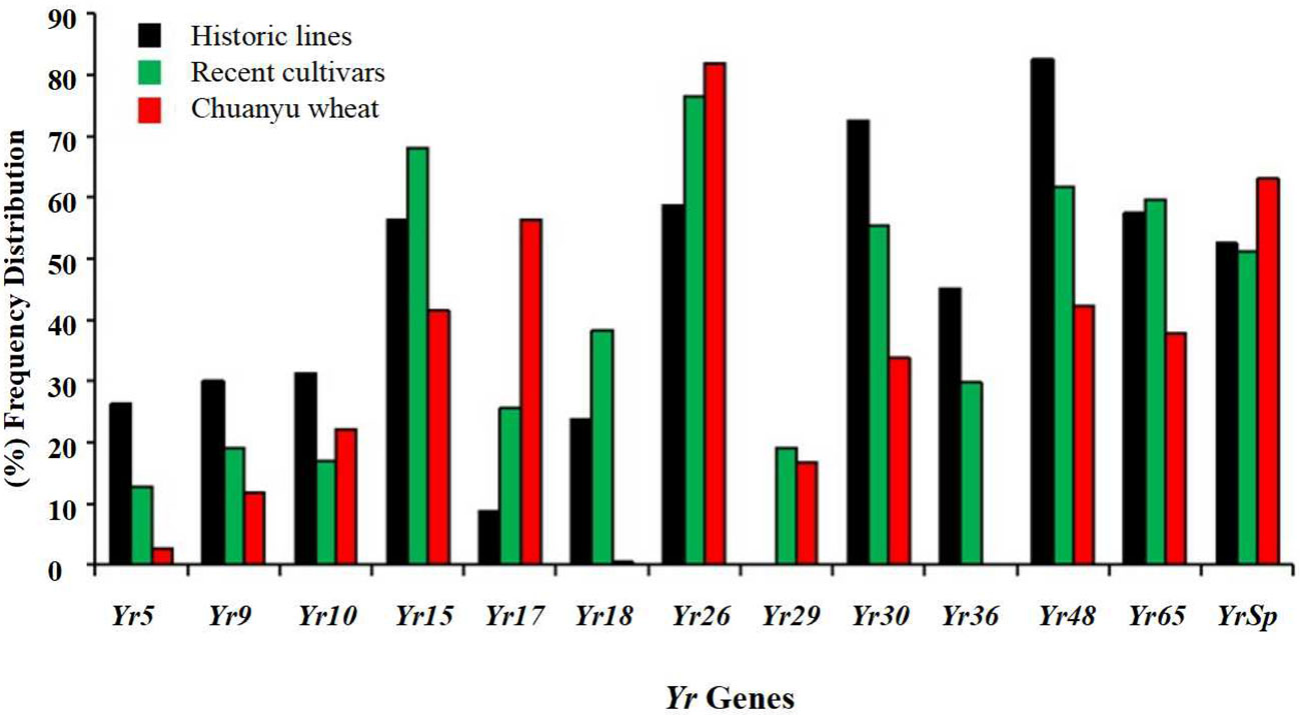
Percentage (%) frequency distribution of 13 major stripe rust resistance genes (*Yr* genes) among three populations. Each bar indicates the percentage of genotypes possessing a particular *Yr* gene.

The present study investigated the distribution of 13 major stripe rust resistance genes, including *Yr5*, *Yr9*, *Yr10*, *Yr15*, *Yr17*, *Yr18*, *Yr26*, *Yr29*, *Yr30*, *Yr36*, *Yr48*, *Yr65*, and *YrSp*. **Figure 4** illustrates the percentage frequency distribution of 13 *Yr* genes. In historic lines of Pakistan, *Yr15*, *Yr26*, *Yr30*, *Yr48*, *Yr65*, and *YrSp* showed its prevalence. Meanwhile, *Yr29* was totally absent, and *Yr17* was present in less than 10% of the population. In recent cultivars of Pakistan, *Yr15* and *Yr26* were overused, and *Yr5*, *Yr9*, and *Yr10* were present in less than 15% of the population. In Chuanyu, wheat *Yr17*, *Yr26*, and *YrSp* were present in more than 50% of the population. *Yr17* was overused in Chuanyu wheat, whereas *Yr18* was rare, and *Yr36* was absent. Stripe rust resistance genes *Yr26*, *Yr15*, and *Yr65* were highly present in all three groups. Detailed information about the presence and absence of genes in each population is mentioned in **Supplementary File S1** (**Supplementary Tables S1**-**S3**). The frequency of each resistance gene across the different wheat groups is in **Supplementary File S1** (**Supplementary Table S5**), which shows the percentage of resistance genes in each population.

### Correlation between the number of pyramided genes and IT value

**Figure 5:**
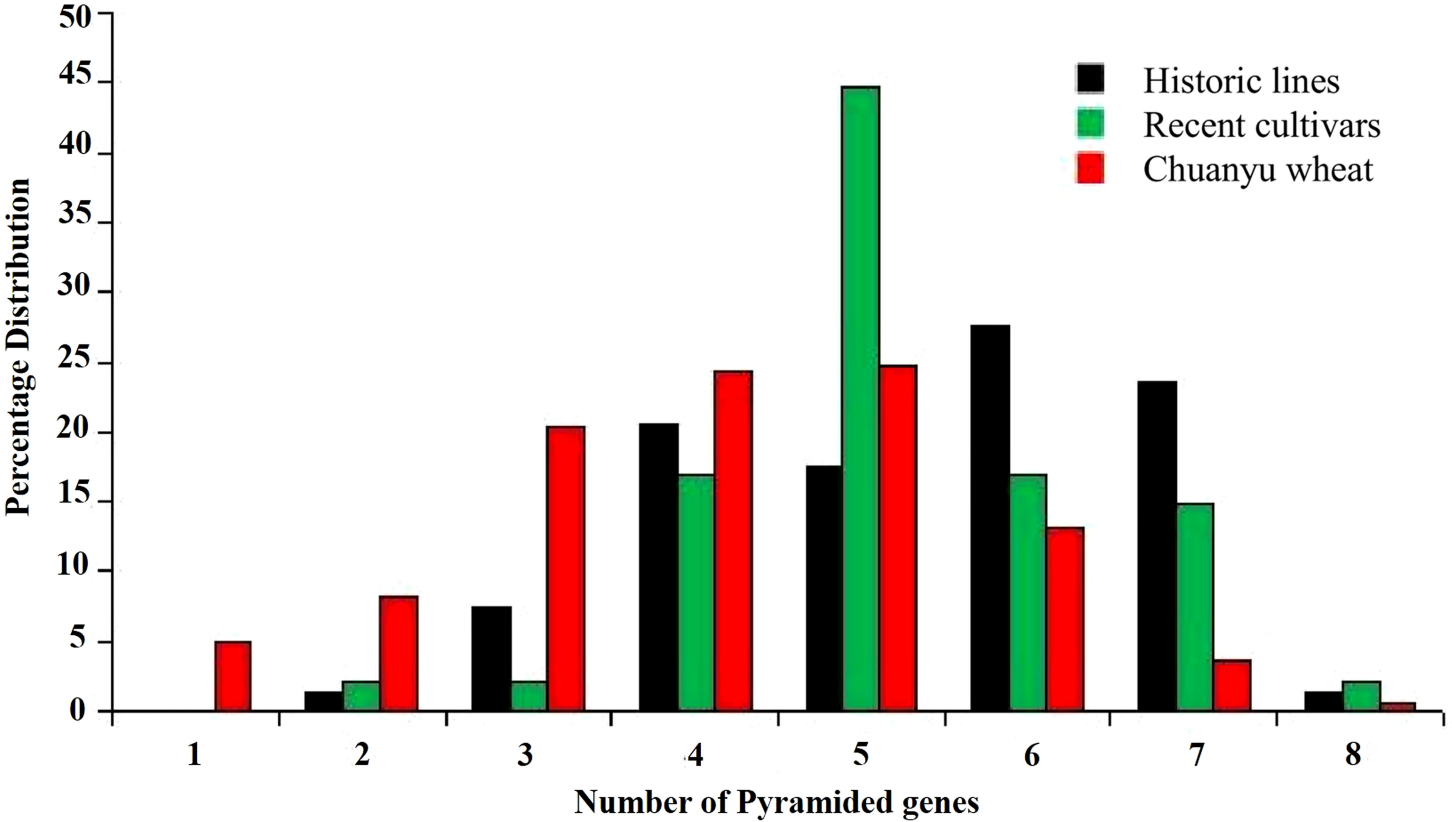
Percentage distribution of pyramided *Yr* genes detected in each population, which illustrates the differences in the frequency of pyramiding in different populations.

A significant number of *Yr* genes are pyramided across all populations. In all three groups, two to eight pyramiding instances of *Yr* genes were observed. In historic lines of Pakistan, the frequency of two and eight pyramided *Yr* genes was less than 3%. In Chuanyu wheat, the number of pyramiding of four, five, and six pyramided *Yr* genes was high, whereas in recent cultivars, the number of five pyramided *Yr* genes was prevalent, whereas four, six, and seven (17%, 11%, and 15%, respectively) pyramided *Yr* genes were observed in recent cultivars. The average number of pyramided *Yr* genes in historic lines and recent cultivars was five genes, whereas in Chuanyu wheat, the average number of pyramided *Yr* genes was four, as shown in **Figure 5**.

**Figure 6:**
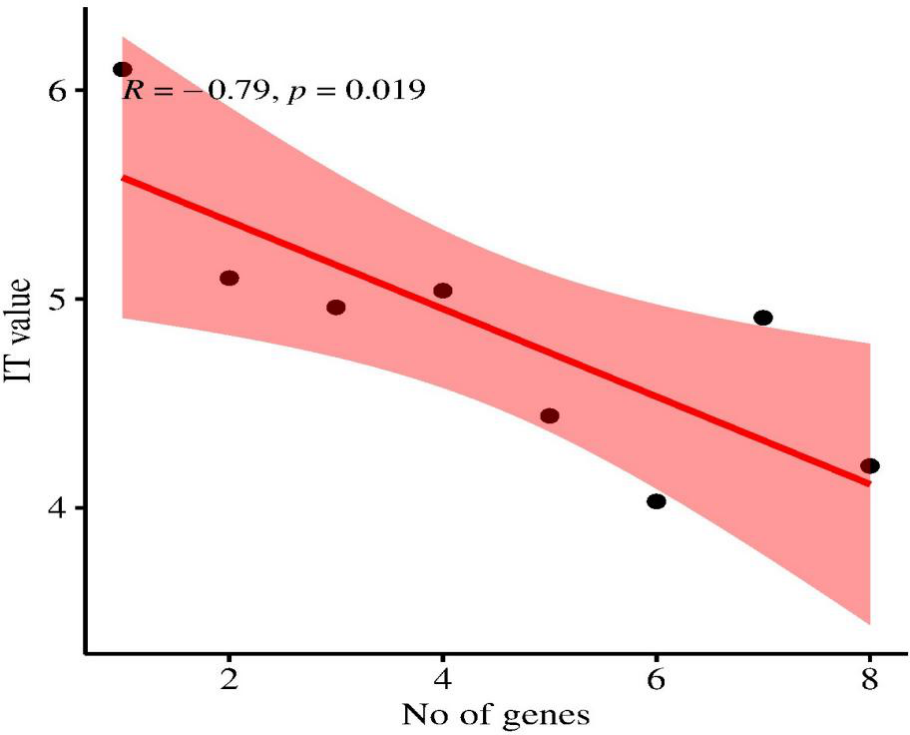
Correlation between the number of *Yr* genes pyramided in wheat genotypes and their corresponding IT (1, 2, 3, 4, 5, 6, 7, 8, and 9) values, showing a significant negative correlation (*r* = -0.79, *P* < 0.01).

Based on the field evaluation of *Pst*, the correlation analysis shows a significant negative correlation between the number of *Yr* genes and IT values, with IT values ranging from 0 to 9. A significant negative correlation (*r* = -0.435, *R* ^2^ = 0.66, and *p* = 0.019) was observed. An increasing number of pyramided *Yr* genes decreases the IT value and increases resistance (**Figure 6**). This suggests that the relationship between *Yr* genes and IT value is statistically significant, indicating that the observed relationship is not likely a result of random chance. Pyramiding effective *Yr* genes in wheat is associated with enhanced stripe rust resistance.

### Effectiveness of single *Yr* gene

**Figure 7:**
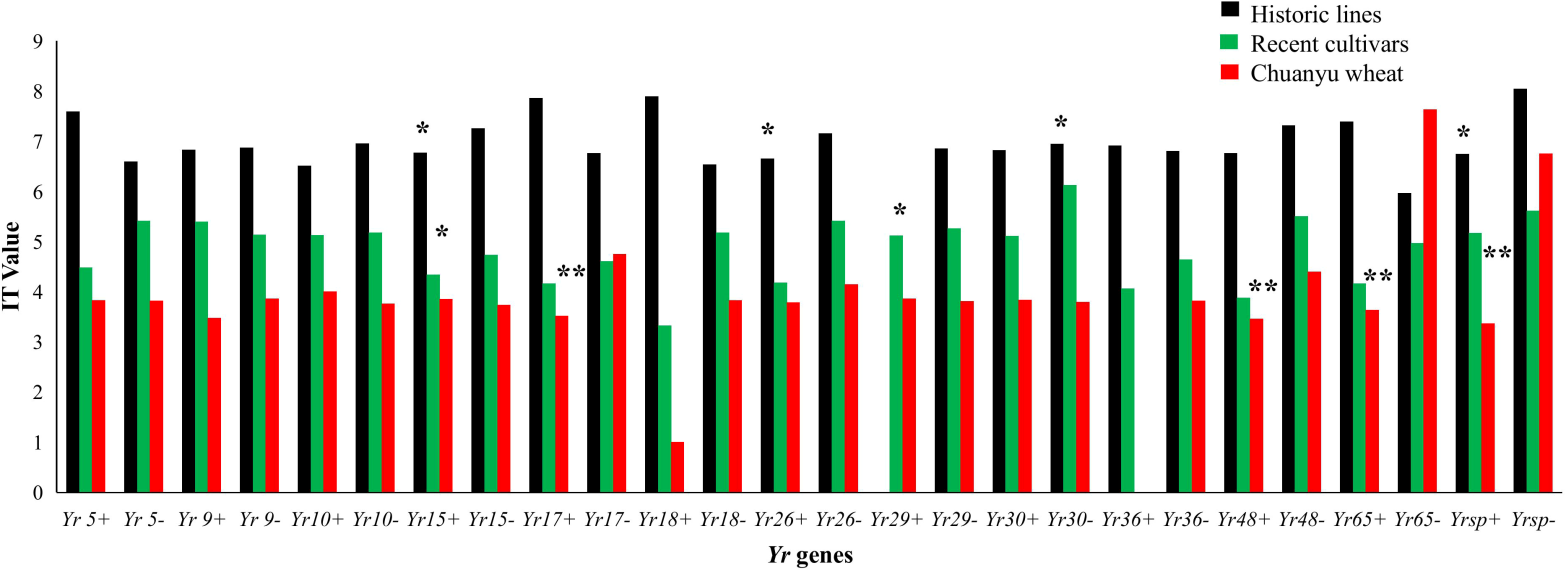
The IT values of each *Yr* gene are compared. Positive markers of the *Yr* gene are indicated by +, while negative markers are indicated by –. Each bar represents the mean value of IT (0, 1, 2, 3, 4, 5, 6, 7, 8, and 9). The significance between each group was tested using one-way ANOVA (*p < 0.05, **p < 0.001).

The stripe rust resistance genes *Yr5* and *Yr15* were effective in historic lines and recent cultivars of Pakistan. The *Yr* genes such as *Yr17*, *Yr48*, and *Yr65* are effective in Chuanyu wheat genotypes (**Figure 7**). *Yr29* likewise showed a substantial increase in effectiveness in recent cultivars of Pakistan. Furthermore, *Yr26* and *Yr30* are effective against current stripe rust races in historic lines of Pakistan. Several resistance genes, including *Yr9*, *Yr10*, *Yr18*, *Yr26*, and *Yr36*, did not show resistance against mixed races during the adult stage.

### Effectiveness of pyramiding *Yr* gene

**Figure 8:**
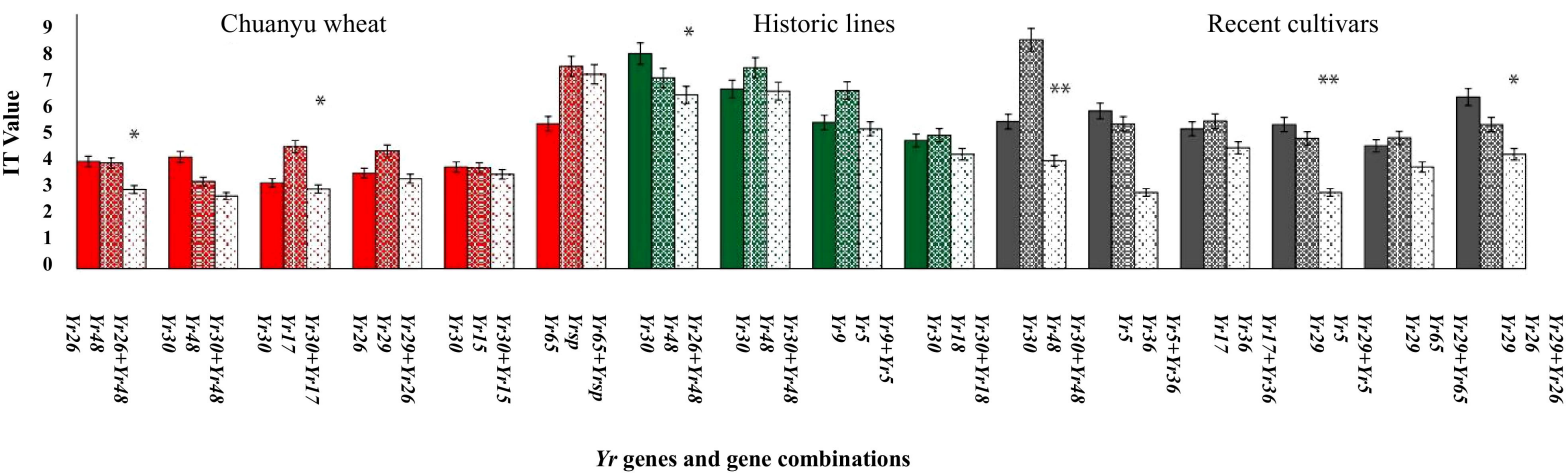
Different combinations of *Yr* genes associated with stripe rust. Each bar represents the mean IT value of stripe rust, ranging from 0 to 9. A one-way ANOVA was conducted to assess the significance among the groups (*p<0.05, **p<0.001).

A total of 64 combinations of two *Yr* genes were analyzed to evaluate the effects of stripe rust in three diverse groups; the results have shown that certain resistance genes displayed epistatic or additive effects when combined (**Figure 8**). The combination of *Yr26+Yr48* and *Yr30+Yr17* in Chuanyu wheat shows a lower IT value (3.1) with a minor standard deviation (1.3) compared to the individual genes alone (*Yr26* IT = 4.2, std = 2.0; *Yr48* IT = 4.2, std = 1.9, *Yr30* 4.4, std = 2.2; *Yr17* IT = 3.1, std = 1.5), suggesting that the pyramided genes are more effective together than separately. Similarly, in Pakistan’s historical lines, the combination of *Yr26+Yr48* shows a significant improvement with an IT, indicating variability in the response but still an overall effective resistance. In recent cultivars of Pakistan, the combination of *Yr29+Yr5* and *Yr30 +Yr48* is marked as significant with an IT of 3.0, suggesting effective resistance. In historical lines, the combination of *Yr30+Yr48* showed no decrease in IT value by adding two genes. If pyramided APR genes are with ASR genes, durable resistance can be achieved. Furthermore, the results suggested an epistatic effect between the pyramided resistance genes, such as in combination with *Yr30+Yr48* in historical lines of Pakistan. Several APR genes, such as *Yr29*, *Yr30*, and *Yr48*, have been discovered to enhance the resistance of specific race-specific ASR genes. These include *Yr5*, *Yr15*, *Yr17*, *Yr64*, and *Yr65* when combined. These findings indicate that these gene combinations hold a significant potential for utilization in resistance breeding. The findings of this study on gene pyramiding in *Yr* will greatly contribute to the development of cultivars with long-lasting resistance.

## Discussion

The constant evolution of the stripe rust races has created challenges in finding effective wheat sources of resistance (Haider et al., 2023). This highlights the need to identify and validate new sources of resistance against the stripe rust pathogen to ensure successful use in breeding programs (Mu et al., 2019). The present study evaluated various genotypes from breeding and genetic programs focused on enhancing wheat resistance to stripe rust in China and Pakistan. In this study, we categorized Pakistani wheat germplasm into two distinct subgroups based on preliminary field evaluation of resistance. The first subgroup consists of genotypes that have significantly lost resistance over time. Conversely, the second subgroup includes genotypes released after 2015, which exhibited noticeably higher levels of resistance in field evaluations (**Figure 3**). This grouping enables a more comprehensive understanding of resistance dynamics over time and assists in the identification of more durable sources of resistance within the recent germplasm.

A total of 349 genotypes from Southwest China and Pakistan were evaluated for stripe rust, and 13 known *Yr* genes were detected—*Yr5*, *Yr9*, *Yr10*, *Yr15*, *Yr17*, *Yr18*, *Yr26*, *Yr29*, *Yr30*, *Yr36*, *Yr48*, *Yr65*, and *YrSp*—using marker-assisted selection (MAS). When performing MAS, it is crucial to consider the credibility and accessibility of the markers (Collard and Mackill, 2007). In this study, functional and linked-based molecular markers were used to identify the corresponding genes. Functional markers and linked markers are effective tools that enable plant breeders to target genes controlling important agronomic traits (Andersen and Lübberstedt, 2003).

The findings from the field evaluation showed different levels of resistance across different wheat groups. Among historic lines of Pakistan, 20% showed resistance, while over 60% were susceptible. For recent cultivars, 45% showed resistance, and 40% to 45% were susceptible. Meanwhile, in Chuanyu wheat, more than 60% exhibited resistance, while 20% to 15% of genotypes showed susceptibility. Certain genotypes such as Pakhtunkhwa-15, Abbasen-21, Wardan 17, Khosha, Nishan, T-9, Dirk, C-228, C-217, Chuanyu 25, Chuanyu29, Chuanyu31, Chuanyu33-1, Chuanyu33-2, Chuanyu37, Chuanyu40, Chuanyu42, Chuanyu45-3, Chuanyu46, and Chuanyu47 exhibited strong resistance to stripe rust, indicating their potential as valuable sources of resistance.

Shahin et al. (2024) employed MAS to identify stripe rust resistance genes in 38 wheat genotypes, including *Yr5*, *Yr9*, *Yr10*, *Yr15*, *Yr17*, *Yr18*, *Yr26*, *Yr29*, *Yr30*, and *Yr36*. They classified these genotypes as resistant and slow-rusting, and they highlighted the significance of pyramiding slow-rusting genes, such as *Yr18* and *Yr29*, to achieve sustainable resistance. The current study investigates the essential role of gene pyramiding in effective resistance; however, the presence of *Yr5*, *Yr10*, and *Yr15* in Pakistani lines shows potential regional differences in the application and effectiveness of resistance genes. Notably, Shahin’s study did not detect *Yr36*, which aligns with findings from Chuanyu wheat, indicating local variations in the utilization of resistance genes and potential resistance on certain genes. The findings of Zheng et al. (2017) support the observations of the current study regarding responses to stripe rust in Chuanyu wheat genotypes. It was found that *Yr18* was rare, and *Yr36* was absent in the Chuanyu wheat genotypes. Chuanyu wheat genotypes have more ASR genes, and some of them were overused, like *Yr17*, *YrSp*, and *Yr48.* The current study suggested that we need to add more APR genes to improve resistance and reduce the overuse of certain *Yr* genes like *Yr17* and *Yr15*. Growing crops with the same resistance genes may have less genetic variety, making them more susceptible to new races. A certain *Pst* race may damage a crop. Overuse of resistance genes can lead to pathogens developing *Pst* races that can overcome current resistance mechanisms (Salgotra and Chauhan, 2023). In historical lines of Pakistan, *Yr15*, *Yr26*, and *YrSp* demonstrated significant effectiveness, showing strong resistance to stripe rust. These findings are consistent with the results of Haider et al. (2023), who proposed the presence of *Yr5*, *Yr10*, *Yr15*, and *Yr26* in Indian varieties. The present study also showed similar results, showing that *Yr* genes such as *Yr5*, *Yr10*, and *Yr*26 exhibited moderate effectiveness, whereas *Yr29* was absent in historical lines.

Pyramiding of effective *Yr* genes increases durable resistance. Molecular markers are useful in identifying wheat genotypes with multiple genes as well as facilitating the pyramiding of resistant genes (Muhammad et al., 2023). The correlation analysis conducted in the current study supports the findings of Liu et al. (2020) on the relationship between the number of pyramided *Yr* genes and IT values. The current study also showed similar results by increasing the number of effective pyramided *Yr* genes. The IT values reduce and enhance resistance to stripe rust.

Moreover, gene combinations like *Yr9+Yr18* and *Yr30+Yr46* have shown significant effects when combined. The results of the current gene pyramiding study is consistent with the findings of Liu et al. (2020), indicating that combining multiple resistance genes can lead to higher levels of resistance than individual genes. This combination can contribute to durable resistance against various diseases—for instance, in Chuanyu wheat, the combination of *Yr26+Yr48* and *Yr30+Yr17* demonstrates a lower IT value (3.1) compared to the individual genes alone. This suggests that pyramiding effective *Yr* genes has a greater effect when used together rather than individually.

Similarly, in Pakistani historic lines and recent cultivars, the combination of *Yr26+Yr48*, *Yr29+Yr5*, and *Yr30+Yr48* is marked as significant and suggests effective resistance. Wang et al. (2022) focused on integrating *Yr18*, *Yr28*, and *Yr36* within a controlled environment, demonstrating additive effects for all-stage resistance. In contrast, the current study evaluated a broader spectrum of genes, including *Yr9*, *Yr26*, *Yr30*, and *Yr48*, across multiple genotypes. Both investigations emphasize that pyramiding multiple genes provides improved and sustained resistance compared to individual genes, with significant implications for advancing global wheat breeding programs. Significant effective genes, such as *Yr15*, *Yr17*, *Yr26*, *Yr48*, *Yr65*, and *YrSp*, will be extensively utilized in breeding programs.

Additionally, genes exhibiting limited effectiveness under current field conditions should still be considered for incorporation in gene pyramiding strategies. This is because a single gene, when used alone, can become susceptible due to genetic shifts in the pathogen (Liu et al., 2020). The current research findings, along with the identification of several quantitative trait loci (QTLs) associated with resistance in wheat, as reported by Rosewarne et al. (2005) and Lowe et al. (2011), suggested that gene pyramiding could greatly enhance the durability of rust resistance. To enhance durable resistance, it is recommended to incorporate APR genes in combination with ASR genes. Furthermore, the results suggest an epistatic effect between the pyramided resistance genes as observed in the combinations of *Yr30+Yr48*, *Yr26+Yr48*, *Yr29+Yr5*, and *Yr30+Yr17*. Using specific combinations of genes for deployment provides long-lasting and enhanced resistance compared to relying on a single gene.

## Conclusion

The findings of the current study emphasize the importance of identifying and pyramiding effective *Yr* genes against stripe rust resistance in wheat genotypes. Specifically, the study highlights the prevalence and effectiveness of certain *Yr* genes, such as *Yr15*, *Yr17*, *Yr26*, *Yr29*, *Yr48*, *Yr65*, and *YrSp*. Current research highlights the significance of pyramiding of effective *Yr* genes and reducing the overuse of certain *Yr* genes. This underscores the significance of gene combination as a strategy to enhance long-lasting resistance to stripe rust. Pakistani breeding programs must incorporate more diverse ASR genes and reduce the overuse of some *Yr* genes like *Yr48*, *Yr65*, and *Yr26*. Chuanyu wheat breeding programs have more ASR genes, but they need to incorporate more APR genes, such as *Yr18* and *Yr36*, to enhance durable resistance.

## Data Availability

The datasets presented in this study can be found in online repositories. The names of the repository/repositories and accession number(s) can be found in the article/**Supplementary Material.**
