# Supplementary material for: Evaluation of stripe rust resistance and analysis of resistance genes in wheat genotypes from Pakistan and Southwest China
Source: Front Plant Sci. 2024 Dec 9;15:1494566. doi: 10.3389/fpls.2024.1494566 (PMC11663667; doi:10.3389/fpls.2024.1494566)
Supplement: Supplementary file 1 [file Table1.docx]

Table S1. Comprehensive information on the phenotypic and genotypic traits of historic wheat lines from Pakistan.

• **S.No**: Genotype identifier.

• **IT Value**: IT value (0-9 scale), with lower scores indicating greater resistance.

• ***Yr* gene** Presence (1) or absence (0) of specific stripe rust resistance genes.

| **S.No** | **IT value** | ***Yr5*** | ***Yr9*** | ***Yr10*** | ***Yr15*** | ***Yr17*** | ***Yr18*** | ***Yr26*** | ***Yr29*** | ***Yr30*** | ***Yr36*** | ***Yr48*** | ***Yr65*** | ***YrSp*** |
| --- | --- | --- | --- | --- | --- | --- | --- | --- | --- | --- | --- | --- | --- | --- |
| G1 | 1 | 0 | 0 | 0 | 1 | 0 | 0 | 0 | 0 | 1 | 1 | 1 | 0 | 1 |
| G2 | 5 | 0 | 1 | 0 | 1 | 0 | 0 | 1 | 0 | 1 | 1 | 1 | 0 | 0 |
| G3 | 2 | 0 | 1 | 0 | 1 | 0 | 0 | 1 | 0 | 1 | 0 | 1 | 0 | 0 |
| G4 | 6 | 1 | 1 | 0 | 1 | 0 | 0 | 1 | 0 | 0 | 0 | 1 | 1 | 1 |
| G5 | 1 | 0 | 1 | 0 | 1 | 0 | 0 | 1 | 0 | 1 | 1 | 1 | 0 | 1 |
| G6 | 0 | 0 | 1 | 0 | 1 | 0 | 0 | 1 | 0 | 1 | 0 | 1 | 0 | 1 |
| G7 | 0 | 0 | 0 | 1 | 1 | 0 | 0 | 1 | 0 | 1 | 0 | 1 | 0 | 1 |
| G8 | 2 | 0 | 0 | 1 | 1 | 0 | 0 | 1 | 0 | 1 | 0 | 1 | 0 | 1 |
| G9 | 9 | 0 | 0 | 1 | 0 | 0 | 0 | 1 | 0 | 1 | 0 | 1 | 0 | 0 |
| G10 | 8 | 1 | 1 | 0 | 0 | 0 | 0 | 1 | 0 | 1 | 0 | 1 | 0 | 0 |
| G11 | 8 | 1 | 0 | 1 | 1 | 0 | 0 | 0 | 0 | 0 | 0 | 1 | 1 | 0 |
| G12 | 7 | 1 | 1 | 1 | 0 | 0 | 0 | 1 | 0 | 1 | 1 | 1 | 1 | 0 |
| G13 | 9 | 0 | 1 | 0 | 0 | 0 | 0 | 0 | 0 | 1 | 0 | 1 | 0 | 0 |
| G14 | 8 | 0 | 1 | 0 | 0 | 0 | 0 | 1 | 0 | 1 | 0 | 1 | 0 | 0 |
| G15 | 6 | 0 | 0 | 1 | 1 | 0 | 0 | 1 | 0 | 1 | 0 | 1 | 0 | 0 |
| G16 | 9 | 0 | 1 | 1 | 0 | 0 | 0 | 1 | 0 | 1 | 1 | 1 | 0 | 1 |
| G17 | 8 | 0 | 0 | 0 | 1 | 0 | 0 | 0 | 0 | 1 | 1 | 1 | 0 | 0 |
| G18 | 7 | 0 | 1 | 0 | 1 | 0 | 1 | 0 | 0 | 1 | 1 | 1 | 0 | 1 |
| G19 | 9 | 0 | 1 | 0 | 0 | 0 | 1 | 1 | 0 | 1 | 1 | 1 | 0 | 0 |
| G20 | 8 | 1 | 0 | 1 | 0 | 1 | 0 | 1 | 0 | 1 | 0 | 1 | 1 | 0 |
| G21 | 2 | 1 | 0 | 1 | 1 | 0 | 0 | 1 | 0 | 0 | 1 | 1 | 0 | 1 |
| G22 | 9 | 1 | 0 | 0 | 0 | 1 | 1 | 0 | 0 | 1 | 1 | 1 | 1 | 0 |
| G23 | 8 | 0 | 1 | 0 | 0 | 0 | 0 | 1 | 0 | 1 | 1 | 1 | 1 | 1 |
| G24 | 8 | 1 | 0 | 0 | 1 | 0 | 0 | 1 | 0 | 1 | 1 | 1 | 0 | 0 |
| G25 | 8 | 0 | 0 | 0 | 1 | 0 | 1 | 1 | 0 | 0 | 0 | 1 | 0 | 1 |
| G26 | 6 | 0 | 1 | 0 | 0 | 0 | 0 | 1 | 0 | 1 | 1 | 1 | 1 | 1 |
| G27 | 6 | 0 | 1 | 0 | 1 | 0 | 0 | 1 | 0 | 0 | 1 | 1 | 1 | 0 |
| G28 | 5 | 0 | 0 | 1 | 1 | 0 | 0 | 1 | 0 | 1 | 1 | 1 | 1 | 0 |
| G29 | 9 | 1 | 0 | 1 | 0 | 0 | 0 | 1 | 0 | 1 | 1 | 1 | 1 | 0 |
| G30 | 8 | 1 | 0 | 1 | 1 | 0 | 1 | 1 | 0 | 1 | 0 | 1 | 0 | 0 |
| G31 | 6 | 0 | 0 | 0 | 1 | 0 | 0 | 1 | 0 | 0 | 1 | 1 | 1 | 1 |
| G32 | 8 | 1 | 0 | 0 | 0 | 0 | 1 | 1 | 0 | 1 | 0 | 0 | 1 | 1 |
| G33 | 9 | 0 | 0 | 0 | 0 | 1 | 0 | 1 | 0 | 1 | 0 | 1 | 0 | 0 |
| G34 | 8 | 0 | 0 | 0 | 0 | 0 | 1 | 1 | 0 | 1 | 0 | 1 | 1 | 1 |
| G35 | 6 | 1 | 1 | 0 | 1 | 0 | 0 | 1 | 0 | 0 | 0 | 1 | 1 | 1 |
| G36 | 7 | 0 | 0 | 0 | 1 | 0 | 0 | 0 | 0 | 1 | 0 | 1 | 1 | 0 |
| G37 | 8 | 0 | 0 | 0 | 0 | 0 | 0 | 0 | 0 | 1 | 0 | 1 | 1 | 1 |
| G38 | 6 | 0 | 0 | 0 | 0 | 0 | 0 | 0 | 0 | 1 | 1 | 1 | 0 | 1 |
| G39 | 7 | 0 | 0 | 1 | 0 | 0 | 0 | 1 | 0 | 0 | 1 | 0 | 0 | 1 |
| G40 | 4 | 0 | 0 | 1 | 1 | 0 | 0 | 0 | 0 | 0 | 0 | 0 | 0 | 0 |
| G41 | 7 | 0 | 0 | 1 | 0 | 0 | 1 | 1 | 0 | 0 | 1 | 1 | 0 | 1 |
| G42 | 8 | 0 | 0 | 0 | 1 | 0 | 0 | 0 | 0 | 1 | 0 | 0 | 0 | 1 |
| G43 | 9 | 0 | 0 | 0 | 0 | 0 | 0 | 1 | 0 | 0 | 1 | 1 | 1 | 0 |
| G44 | 7 | 0 | 0 | 1 | 1 | 1 | 0 | 1 | 0 | 0 | 1 | 1 | 1 | 1 |
| G45 | 8 | 0 | 0 | 0 | 0 | 0 | 0 | 0 | 0 | 1 | 0 | 1 | 1 | 0 |
| G46 | 8 | 0 | 0 | 0 | 0 | 0 | 0 | 1 | 0 | 1 | 0 | 0 | 0 | 1 |
| G47 | 8 | 0 | 0 | 0 | 1 | 0 | 0 | 0 | 0 | 1 | 0 | 1 | 1 | 1 |
| G48 | 7 | 0 | 0 | 0 | 1 | 0 | 0 | 1 | 0 | 1 | 0 | 1 | 0 | 1 |
| G49 | 6 | 0 | 0 | 1 | 1 | 0 | 0 | 0 | 0 | 0 | 0 | 1 | 1 | 0 |
| G50 | 7 | 1 | 0 | 0 | 1 | 0 | 0 | 1 | 0 | 1 | 0 | 1 | 1 | 0 |
| G51 | 8 | 0 | 0 | 0 | 0 | 0 | 0 | 1 | 0 | 0 | 0 | 0 | 1 | 1 |
| G52 | 8 | 1 | 1 | 0 | 1 | 0 | 0 | 0 | 0 | 1 | 0 | 0 | 1 | 1 |
| G53 | 5 | 0 | 0 | 0 | 1 | 0 | 0 | 0 | 0 | 0 | 0 | 1 | 1 | 1 |
| G54 | 8 | 0 | 0 | 0 | 0 | 0 | 0 | 1 | 0 | 0 | 1 | 1 | 1 | 0 |
| G55 | 7 | 0 | 0 | 0 | 1 | 0 | 0 | 0 | 0 | 0 | 1 | 1 | 1 | 1 |
| G56 | 6 | 0 | 0 | 0 | 1 | 0 | 0 | 1 | 0 | 0 | 1 | 1 | 1 | 1 |
| G57 | 8 | 0 | 0 | 0 | 0 | 0 | 0 | 1 | 0 | 1 | 1 | 0 | 1 | 0 |
| G58 | 7 | 0 | 0 | 0 | 1 | 0 | 0 | 1 | 0 | 0 | 1 | 1 | 1 | 1 |
| G59 | 8 | 0 | 0 | 1 | 0 | 0 | 0 | 0 | 0 | 1 | 0 | 1 | 1 | 0 |
| G60 | 8 | 0 | 0 | 1 | 1 | 0 | 1 | 0 | 0 | 1 | 0 | 1 | 1 | 1 |
| G61 | 9 | 1 | 1 | 0 | 0 | 0 | 0 | 0 | 0 | 1 | 1 | 1 | 1 | 0 |
| G62 | 9 | 1 | 0 | 0 | 0 | 0 | 1 | 0 | 0 | 1 | 1 | 1 | 1 | 0 |
| G63 | 8 | 0 | 0 | 0 | 0 | 0 | 1 | 1 | 0 | 1 | 1 | 0 | 1 | 1 |
| G64 | 7 | 1 | 0 | 0 | 1 | 1 | 0 | 0 | 0 | 1 | 0 | 0 | 0 | 0 |
| G65 | 5 | 0 | 0 | 0 | 1 | 0 | 0 | 0 | 0 | 1 | 1 | 0 | 1 | 1 |
| G66 | 6 | 0 | 0 | 1 | 0 | 0 | 1 | 0 | 0 | 1 | 1 | 1 | 0 | 1 |
| G67 | 8 | 0 | 0 | 0 | 1 | 0 | 0 | 0 | 0 | 0 | 1 | 1 | 1 | 0 |
| G68 | 9 | 0 | 1 | 0 | 0 | 1 | 1 | 0 | 0 | 1 | 0 | 1 | 0 | 0 |
| G69 | 8 | 0 | 1 | 0 | 1 | 0 | 0 | 1 | 0 | 1 | 0 | 1 | 1 | 1 |
| G70 | 8 | 1 | 0 | 1 | 1 | 0 | 1 | 0 | 0 | 1 | 0 | 0 | 0 | 1 |
| G71 | 8 | 1 | 0 | 0 | 0 | 0 | 1 | 0 | 0 | 1 | 0 | 1 | 1 | 1 |
| G72 | 8 | 0 | 0 | 1 | 1 | 0 | 1 | 0 | 0 | 1 | 0 | 1 | 1 | 1 |
| G73 | 6 | 0 | 1 | 1 | 1 | 0 | 0 | 0 | 0 | 1 | 0 | 1 | 1 | 0 |
| G74 | 7 | 0 | 0 | 0 | 1 | 0 | 0 | 0 | 0 | 1 | 0 | 1 | 1 | 1 |
| G75 | 7 | 0 | 0 | 0 | 1 | 1 | 0 | 0 | 0 | 0 | 1 | 1 | 0 | 1 |
| G76 | 8 | 0 | 0 | 0 | 0 | 0 | 1 | 0 | 0 | 1 | 0 | 0 | 1 | 0 |
| G77 | 8 | 1 | 1 | 0 | 0 | 0 | 1 | 1 | 0 | 1 | 1 | 1 | 0 | 0 |
| G78 | 6 | 0 | 0 | 1 | 1 | 0 | 0 | 1 | 0 | 1 | 0 | 1 | 1 | 1 |
| G79 | 9 | 0 | 1 | 0 | 0 | 0 | 0 | 1 | 0 | 0 | 1 | 1 | 1 | 0 |
| G80 | 9 | 1 | 1 | 1 | 0 | 0 | 1 | 1 | 0 | 1 | 0 | 0 | 1 | 0 |

Table S2. Comprehensive information on the phenotypic and genotypic traits of recent cultivars from Pakistan**.**

• **S.No**: Genotype identifier.

• **IT value**: IT value (0-9 scale), with lower scores indicating greater resistance.

• ***Yr* gene** Presence (1) or absence (0) of specific stripe rust resistance genes.

| **S.No** | **IT value** | ***Yr5*** | ***Yr9*** | ***Yr10*** | ***Yr15*** | ***Yr17*** | ***Yr18*** | ***Yr26*** | ***Yr29*** | ***Yr30*** | ***Yr36*** | ***Yr48*** | ***Yr65*** | ***YrSp*** |
| --- | --- | --- | --- | --- | --- | --- | --- | --- | --- | --- | --- | --- | --- | --- |
| D1 | 5 | 0 | 0 | 0 | 1 | 0 | 0 | 1 | 0 | 1 | 1 | 0 | 0 | 1 |
| D2 | 7 | 0 | 1 | 0 | 1 | 0 | 0 | 1 | 0 | 0 | 0 | 0 | 0 | 1 |
| D3 | 7 | 1 | 0 | 1 | 0 | 0 | 0 | 0 | 0 | 1 | 0 | 0 | 0 | 1 |
| D4 | 3 | 1 | 0 | 1 | 1 | 0 | 1 | 1 | 0 | 1 | 0 | 1 | 0 | 1 |
| D5 | 5 | 0 | 0 | 0 | 1 | 0 | 0 | 0 | 0 | 1 | 0 | 1 | 1 | 1 |
| D6 | 6 | 0 | 0 | 1 | 1 | 0 | 1 | 0 | 0 | 1 | 0 | 1 | 1 | 0 |
| D7 | 4 | 1 | 1 | 0 | 1 | 0 | 0 | 1 | 0 | 1 | 0 | 1 | 0 | 1 |
| D8 | 5 | 0 | 1 | 0 | 1 | 0 | 0 | 0 | 0 | 1 | 0 | 0 | 1 | 1 |
| D9 | 7 | 0 | 0 | 1 | 1 | 0 | 0 | 1 | 0 | 1 | 1 | 0 | 1 | 1 |
| D10 | 6 | 0 | 0 | 0 | 1 | 0 | 1 | 0 | 0 | 1 | 0 | 0 | 1 | 1 |
| D11 | 7 | 0 | 0 | 0 | 1 | 0 | 0 | 1 | 0 | 1 | 1 | 0 | 1 | 0 |
| D12 | 5 | 0 | 1 | 0 | 1 | 1 | 1 | 1 | 0 | 1 | 0 | 0 | 0 | 0 |
| D13 | 4 | 0 | 0 | 1 | 1 | 1 | 0 | 0 | 0 | 1 | 1 | 0 | 1 | 1 |
| D14 | 5 | 1 | 0 | 0 | 1 | 0 | 0 | 1 | 0 | 1 | 0 | 0 | 1 | 0 |
| D15 | 6 | 0 | 1 | 1 | 0 | 1 | 0 | 1 | 1 | 0 | 0 | 0 | 1 | 1 |
| D16 | 6 | 0 | 1 | 1 | 1 | 0 | 0 | 1 | 1 | 1 | 0 | 1 | 0 | 1 |
| D17 | 7 | 0 | 0 | 0 | 0 | 0 | 0 | 0 | 1 | 1 | 0 | 0 | 0 | 1 |
| D18 | 8 | 0 | 0 | 0 | 0 | 1 | 0 | 1 | 1 | 1 | 0 | 0 | 0 | 1 |
| D19 | 7 | 0 | 1 | 0 | 0 | 0 | 0 | 0 | 0 | 1 | 0 | 1 | 0 | 1 |
| D20 | 4 | 0 | 0 | 0 | 0 | 0 | 0 | 1 | 1 | 1 | 0 | 1 | 0 | 1 |
| D21 | 4 | 0 | 0 | 0 | 0 | 1 | 0 | 0 | 1 | 1 | 0 | 1 | 1 | 1 |
| D22 | 6 | 0 | 1 | 0 | 0 | 1 | 0 | 0 | 1 | 0 | 0 | 1 | 0 | 1 |
| D23 | 4 | 1 | 1 | 0 | 0 | 1 | 0 | 1 | 1 | 1 | 1 | 0 | 0 | 1 |
| D24 | 2 | 0 | 0 | 0 | 0 | 0 | 0 | 1 | 1 | 1 | 0 | 1 | 0 | 1 |
| D25 | 4 | 1 | 0 | 0 | 0 | 0 | 0 | 1 | 0 | 1 | 0 | 1 | 0 | 1 |
| D26 | 6 | 0 | 0 | 0 | 1 | 0 | 0 | 1 | 0 | 1 | 0 | 1 | 0 | 1 |
| D27 | 5 | 0 | 0 | 0 | 1 | 1 | 0 | 1 | 0 | 1 | 0 | 0 | 0 | 0 |
| D28 | 3 | 0 | 0 | 1 | 0 | 0 | 0 | 0 | 0 | 1 | 0 | 0 | 1 | 1 |
| D29 | 5 | 0 | 0 | 0 | 0 | 0 | 0 | 1 | 0 | 1 | 0 | 0 | 1 | 1 |
| D30 | 3 | 0 | - | - | 1 | 0 | 1 | 1 | - | - | 1 | 1 | 0 | - |
| D31 | 5 | 0 | - | - | 1 | 0 | 1 | 1 | - | - | 1 | 1 | 1 | - |
| D32 | 6 | 0 | - | - | 1 | 0 | 1 | 1 | - | - | 1 | 1 | 1 | - |
| D33 | 5 | 0 | - | - | 1 | 0 | 0 | 1 | - | - | 0 | 1 | 1 | - |
| D34 | 6 | 0 | - | - | 1 | 0 | 0 | 1 | - | - | 0 | 1 | 1 | - |
| D35 | 5 | 0 | - | - | 1 | 0 | 0 | 1 | - | - | 1 | 1 | 1 | - |
| D36 | 6 | 0 | - | - | 1 | 0 | 1 | 1 | - | - | 1 | 1 | 1 | - |
| D37 | 3 | 0 | - | - | 1 | 0 | 1 | 1 | - | - | 0 | 1 | 1 | - |
| D38 | 2 | 0 | - | - | 1 | 1 | 1 | 1 | - | - | 0 | 1 | 0 | - |
| D39 | 3 | 0 | - | - | 1 | 1 | 1 | 1 | - | - | 1 | 0 | 1 | - |
| D40 | 3 | 0 | - | - | 1 | 0 | 1 | 1 | - | - | 0 | 1 | 1 | - |
| D41 | 4 | 0 | - | - | 1 | 1 | 1 | 1 | - | - | 0 | 1 | 1 | - |
| D42 | 0 | 0 | - | - | 1 | 0 | 1 | 1 | - | - | 0 | 1 | 1 | - |
| D43 | 3 | 0 | - | - | 1 | 0 | 1 | 1 | - | - | 0 | 1 | 1 | - |
| D44 | 3 | 0 | - | - | 1 | 0 | 1 | 1 | - | - | 0 | 1 | 1 | - |
| D45 | 1 | 0 | - | - | 1 | 1 | 1 | 1 | - | - | 1 | 1 | 1 | - |
| D46 | 3 | 0 | - | - | 0 | 0 | 0 | 1 | - | - | 1 | 0 | 0 | - |
| D47 | 1 | 0 | - | - | 0 | 0 | 1 | 1 | - | - | 1 | 1 | 1 | - |

Table S3. Present the phenotypic and genotypic characteristics of Chuanyu wheat genotypes

• **S.No**: Genotype identifier.

• **IT Value**: IT value (0-9 scale), with lower scores indicating greater resistance.

• ***Yr* gene** Presence (1) or absence (0) of specific stripe rust resistance genes.

| **S.**  **No** | **IT**  **value** | ***Yr5*** | ***Yr9*** | ***Yr10*** | ***Yr15*** | ***Yr17*** | ***Yr18*** | ***Yr26*** | ***Yr29*** | ***Yr30*** | ***Yr36*** | ***Yr48*** | ***Yr65*** | | ***YrSp*** |
| --- | --- | --- | --- | --- | --- | --- | --- | --- | --- | --- | --- | --- | --- | --- | --- |
| R1 | 5 | 0 | 0 | 0 | 0 | 1 | 0 | 0 | 1 | 0 | 0 | 1 | 0 | 0 | |
| R2 | 9 | 0 | 0 | 0 | 0 | 0 | 0 | 0 | 1 | 0 | 0 | 1 | 0 | 1 | |
| R3 | 4 | 0 | 0 | 0 | 0 | 0 | 0 | 1 | 1 | 0 | 0 | 0 | 0 | 1 | |
| R4 | 8 | 0 | 0 | 0 | 0 | 0 | 0 | 1 | 0 | 1 | 0 | 0 | 0 | 0 | |
| R5 | 3 | 0 | 0 | 0 | 0 | 1 | 0 | 1 | 0 | 0 | 0 | 1 | 0 | 1 | |
| R6 | 7 | 0 | 0 | 0 | 0 | 0 | 0 | 1 | 0 | 1 | 0 | 0 | 0 | 0 | |
| R7 | 5 | 0 | 0 | 0 | 0 | 0 | 0 | 1 | 0 | 1 | 0 | 0 | 0 | 1 | |
| R8 | 1 | 0 | 0 | 0 | 1 | 1 | 0 | 1 | 0 | 1 | 0 | 0 | 1 | 1 | |
| R9 | 3 | 0 | 0 | 0 | 0 | 1 | 0 | 0 | 0 | 1 | 0 | 0 | 0 | 1 | |
| R10 | 4 | 0 | 0 | 0 | 0 | 1 | 0 | 1 | 0 | 1 | 0 | 0 | 1 | 0 | |
| R11 | 3 | 0 | 0 | 0 | 1 | 1 | 0 | 1 | 1 | 0 | 0 | 0 | 0 | 1 | |
| R12 | 3 | 0 | 0 | 0 | 1 | 1 | 0 | 1 | 1 | 1 | 0 | 0 | 1 | 1 | |
| R13 | 4 | 0 | 0 | 1 | 0 | 0 | 0 | 0 | 1 | 0 | 0 | 1 | 0 | 1 | |
| R14 | 4 | 0 | 0 | 0 | 0 | 0 | 0 | 0 | 1 | 0 | 0 | 1 | 0 | 0 | |
| R15 | 5 | 0 | 1 | 0 | 0 | 0 | 0 | 0 | 1 | 0 | 0 | 0 | 0 | 0 | |
| R16 | 3 | 0 | 0 | 0 | 0 | 0 | 0 | 1 | 1 | 0 | 0 | 1 | 1 | 1 | |
| R17 | 5 | 0 | 0 | 0 | 1 | 1 | 0 | 1 | 0 | 1 | 0 | 1 | 0 | 0 | |
| R18 | 1 | 0 | 0 | 0 | 0 | 1 | 0 | 1 | 0 | 0 | 0 | 1 | 1 | 1 | |
| R19 | 1 | 0 | 0 | 0 | 1 | 1 | 1 | 1 | 0 | 1 | 0 | 1 | 0 | 0 | |
| R20 | 4 | 0 | 0 | 0 | 1 | 0 | 0 | 0 | 1 | 0 | 0 | 0 | 1 | 1 | |
| R21 | 4 | 1 | 0 | 0 | 1 | 1 | 0 | 0 | 0 | 1 | 0 | 0 | 0 | 1 | |
| R22 | 3 | 0 | 0 | 0 | 1 | 1 | 0 | 1 | 0 | 1 | 0 | 1 | 1 | 0 | |
| R23 | 3 | 0 | 0 | 0 | 0 | 1 | 0 | 1 | 0 | 0 | 0 | 1 | 0 | 1 | |
| R24 | 3 | 0 | 0 | 0 | 0 | 1 | 0 | 1 | 0 | 1 | 0 | 0 | 0 | 0 | |
| R25 | 3 | 0 | 1 | 0 | 0 | 0 | 0 | 0 | 0 | 1 | 0 | 0 | 1 | 1 | |
| R26 | 3 | 0 | 1 | 1 | 0 | 1 | 0 | 0 | 0 | 0 | 0 | 1 | 0 | 0 | |
| R27 | 6 | 0 | 1 | 0 | 1 | 0 | 0 | 1 | 0 | 0 | 0 | 1 | 0 | 0 | |
| R28 | 3 | 0 | 1 | 1 | 1 | 1 | 0 | 1 | 0 | 0 | 0 | 1 | 1 | 1 | |
| R29 | 3 | 0 | 0 | 1 | 0 | 1 | 0 | 1 | 1 | 0 | 0 | 1 | 1 | 0 | |
| R30 | 4 | 0 | 0 | 0 | 0 | 1 | 0 | 1 | 0 | 0 | 0 | 0 | 0 | 1 | |
| R31 | 4 | 0 | 0 | 1 | 1 | 1 | 0 | 1 | 0 | 0 | 0 | 0 | 0 | 1 | |
| R32 | 1 | 0 | 0 | 0 | 0 | 0 | 0 | 1 | 0 | 0 | 0 | 1 | 1 | 0 | |
| R33 | 1 | 0 | 0 | 0 | 0 | 1 | 0 | 1 | 0 | 1 | 0 | 0 | 1 | 1 | |
| R34 | 1 | 0 | 0 | 0 | 1 | 0 | 0 | 1 | 0 | 1 | 0 | 1 | 1 | 1 | |
| R35 | 5 | 0 | 0 | 0 | 1 | 1 | 0 | 1 | 0 | 0 | 0 | 0 | 0 | 0 | |
| R36 | 3 | 0 | 0 | 0 | 0 | 1 | 0 | 1 | 0 | 0 | 0 | 0 | 0 | 1 | |
| R37 | 1 | 0 | 0 | 1 | 1 | 0 | 0 | 1 | 0 | 1 | 0 | 1 | 0 | 1 | |
| R38 | 4 | 0 | 0 | 1 | 0 | 1 | 0 | 0 | 0 | 0 | 0 | 1 | 0 | 1 | |
| R39 | 3 | 0 | 0 | 0 | 1 | 1 | 0 | 0 | 1 | 1 | 0 | 1 | 1 | 1 | |
| R40 | 3 | 0 | 0 | 0 | 0 | 1 | 0 | 1 | 0 | 0 | 0 | 1 | 0 | 1 | |
| R41 | 1 | 1 | 0 | 0 | 0 | 1 | 0 | 1 | 0 | 1 | 0 | 0 | 0 | 0 | |
| R42 | 1 | 0 | 0 | 0 | 1 | 1 | 0 | 0 | 1 | 1 | 0 | 0 | 1 | 0 | |
| R43 | 5 | 0 | 0 | 0 | 1 | 0 | 0 | 0 | 1 | 1 | 0 | 0 | 0 | 0 | |
| R44 | 4 | 0 | 0 | 1 | 1 | 1 | 0 | 1 | 1 | 0 | 0 | 0 | 0 | 1 | |
| R45 | 4 | 0 | 0 | 1 | 1 | 0 | 0 | 1 | 0 | 1 | 0 | 1 | 0 | 1 | |
| R46 | 4 | 0 | 0 | 0 | 0 | 1 | 0 | 1 | 1 | 1 | 0 | 1 | 0 | 0 | |
| R47 | 5 | 0 | 1 | 0 | 1 | 1 | 0 | 0 | 0 | 0 | 0 | 0 | 1 | 1 | |
| R48 | 6 | 0 | 1 | 0 | 0 | 0 | 0 | 0 | 0 | 0 | 0 | 0 | 0 | 0 | |
| R49 | 6 | 0 | 0 | 0 | 1 | 0 | 0 | 1 | 0 | 0 | 0 | 0 | 0 | 0 | |
| R50 | 1 | 0 | 0 | 0 | 0 | 1 | 0 | 1 | 0 | 1 | 0 | 0 | 1 | 1 | |
| R51 | 4 | 0 | 0 | 1 | 1 | 1 | 0 | 1 | 1 | 0 | 0 | 1 | 0 | 0 | |
| R52 | 1 | 0 | 0 | 0 | 1 | 0 | 0 | 1 | 0 | 0 | 0 | 0 | 0 | 1 | |
| R53 | 1 | 0 | 0 | 0 | 0 | 1 | 0 | 1 | 1 | 0 | 0 | 0 | 1 | 1 | |
| R54 | 6 | 0 | 0 | 0 | 1 | 1 | 0 | 0 | 1 | 0 | 0 | 1 | 0 | 0 | |
| R55 | 6 | 0 | 0 | 0 | 1 | 0 | 0 | 1 | 1 | 0 | 0 | 0 | 0 | 0 | |
| R56 | 3 | 0 | 0 | 0 | 1 | 0 | 0 | 1 | 0 | 0 | 0 | 0 | 0 | 1 | |
| R57 | 3 | 0 | 0 | 1 | 0 | 1 | 0 | 1 | 1 | 1 | 0 | 0 | 0 | 0 | |
| R58 | 4 | 0 | 0 | 1 | 1 | 1 | 0 | 1 | 0 | 0 | 0 | 1 | 0 | 1 | |
| R59 | 1 | 0 | 0 | 0 | 1 | 0 | 0 | 1 | 0 | 1 | 0 | 0 | 0 | 1 | |
| R60 | 3 | 0 | 0 | 0 | 1 | 0 | 0 | 1 | 0 | 1 | 0 | 1 | 0 | 1 | |
| R61 | 5 | 0 | 0 | 1 | 0 | 1 | 0 | 1 | 0 | 0 | 0 | 1 | 1 | 1 | |
| R62 | 6 | 0 | 0 | 1 | 0 | 1 | 0 | 1 | 1 | 1 | 0 | 0 | 0 | 0 | |
| R63 | 4 | 0 | 0 | 1 | 1 | 0 | 0 | 1 | 0 | 0 | 0 | 0 | 0 | 0 | |
| R64 | 4 | 0 | 0 | 0 | 0 | 1 | 0 | 0 | 0 | 0 | 0 | 1 | 1 | 0 | |
| R65 | 3 | 0 | 0 | 0 | 1 | 1 | 0 | 1 | 1 | 0 | 0 | 1 | 0 | 0 | |
| R66 | 3 | 0 | 0 | 1 | 0 | 1 | 0 | 0 | 0 | 0 | 0 | 1 | 1 | 0 | |
| R67 | 5 | 0 | 0 | 0 | 0 | 0 | 0 | 1 | 0 | 0 | 0 | 0 | 0 | 0 | |
| R68 | 4 | 0 | 0 | 0 | 0 | 0 | 0 | 1 | 0 | 1 | 0 | 0 | 1 | 1 | |
| R69 | 3 | 0 | 0 | 0 | 0 | 1 | 0 | 1 | 0 | 1 | 0 | 0 | 1 | 0 | |
| R70 | 3 | 0 | 0 | 0 | 1 | 1 | 0 | 1 | 0 | 1 | 0 | 1 | 0 | 1 | |
| R71 | 3 | 0 | 0 | 0 | 0 | 1 | 0 | 1 | 0 | 1 | 0 | 1 | 1 | 0 | |
| R72 | 5 | 0 | 0 | 1 | 1 | 0 | 0 | 1 | 1 | 0 | 0 | 0 | 0 | 1 | |
| R73 | 1 | 0 | 0 | 1 | 0 | 0 | 0 | 0 | 0 | 0 | 0 | 0 | 1 | 1 | |
| R74 | 5 | 0 | 0 | 1 | 0 | 1 | 0 | 0 | 1 | 1 | 0 | 0 | 0 | 1 | |
| R75 | 4 | 0 | 0 | 1 | 0 | 1 | 0 | 1 | 1 | 0 | 0 | 1 | 1 | 1 | |
| R76 | 4 | 0 | 0 | 1 | 1 | 1 | 0 | 1 | 0 | 0 | 0 | 1 | 0 | 1 | |
| R77 | 1 | 0 | 0 | 0 | 1 | 1 | 0 | 1 | 1 | 0 | 0 | 1 | 1 | 1 | |
| R78 | 1 | 0 | 0 | 0 | 1 | 0 | 0 | 1 | 1 | 0 | 0 | 1 | 1 | 1 | |
| R79 | 3 | 0 | 0 | 0 | 0 | 1 | 0 | 1 | 1 | 0 | 0 | 1 | 1 | 1 | |
| R80 | 7 | 0 | 0 | 1 | 0 | 0 | 0 | 1 | 1 | 0 | 0 | 0 | 0 | 0 | |
| R81 | 6 | 0 | 0 | 1 | 1 | 0 | 0 | 1 | 1 | 1 | 0 | 0 | 0 | 0 | |
| R82 | 1 | 0 | 0 | 0 | 0 | 0 | 0 | 1 | 1 | 1 | 0 | 1 | 1 | 1 | |
| R83 | 5 | 0 | 0 | 0 | 0 | 0 | 0 | 1 | 1 | 1 | 0 | 0 | 1 | 1 | |
| R84 | 5 | 0 | 0 | 0 | 1 | 0 | 0 | 1 | 0 | 1 | 0 | 0 | 0 | 1 | |
| R85 | 1 | 0 | 1 | 0 | 0 | 1 | 0 | 0 | 0 | 0 | 0 | 1 | 1 | 1 | |
| R86 | 1 | 0 | 1 | 0 | 1 | 0 | 0 | 1 | 0 | 0 | 0 | 1 | 1 | 1 | |
| R87 | 4 | 0 | 1 | 0 | 0 | 1 | 0 | 1 | 0 | 0 | 0 | 1 | 0 | 1 | |
| R88 | 4 | 0 | 1 | 0 | 0 | 1 | 0 | 1 | 0 | 0 | 0 | 1 | 0 | 1 | |
| R89 | 3 | 0 | 0 | 0 | 1 | 0 | 0 | 1 | 1 | 1 | 0 | 1 | 1 | 1 | |
| R90 | 6 | 0 | 0 | 0 | 0 | 0 | 0 | 1 | 0 | 0 | 0 | 0 | 0 | 0 | |
| R91 | 7 | 0 | 0 | 0 | 1 | 0 | 0 | 1 | 0 | 1 | 0 | 0 | 0 | 0 | |
| R92 | 6 | 0 | 0 | 0 | 0 | 0 | 0 | 1 | 0 | 0 | 0 | 0 | 0 | 0 | |
| R93 | 5 | 0 | 0 | 0 | 0 | 1 | 0 | 1 | 0 | 1 | 0 | 0 | 1 | 1 | |
| R94 | 5 | 0 | 0 | 0 | 0 | 1 | 0 | 1 | 0 | 0 | 0 | 0 | 0 | 1 | |
| R95 | 3 | 0 | 0 | 0 | 1 | 1 | 0 | 1 | 0 | 0 | 0 | 1 | 0 | 1 | |
| R96 | 1 | 0 | 0 | 1 | 1 | 1 | 0 | 1 | 1 | 0 | 0 | 0 | 1 | 0 | |
| R97 | 5 | 0 | 0 | 0 | 0 | 1 | 0 | 1 | 0 | 0 | 0 | 0 | 0 | 1 | |
| R98 | 3 | 0 | 0 | 0 | 1 | 1 | 0 | 1 | 0 | 1 | 0 | 0 | 0 | 1 | |
| R99 | 7 | 0 | 0 | 0 | 0 | 0 | 0 | 1 | 0 | 0 | 0 | 0 | 0 | 0 | |
| R100 | 7 | 0 | 0 | 0 | 1 | 0 | 0 | 0 | 0 | 0 | 0 | 0 | 0 | 0 | |
| R101 | 6 | 0 | 0 | 0 | 1 | 0 | 0 | 0 | 0 | 0 | 0 | 0 | 0 | 0 | |
| R102 | 5 | 0 | 0 | 0 | 0 | 1 | 0 | 1 | 0 | 0 | 0 | 1 | 1 | 1 | |
| R103 | 3 | 0 | 0 | 0 | 0 | 1 | 0 | 1 | 0 | 0 | 0 | 1 | 0 | 1 | |
| R104 | 4 | 0 | 0 | 0 | 1 | 1 | 0 | 1 | 0 | 0 | 0 | 0 | 0 | 1 | |
| R105 | 3 | 0 | 0 | 0 | 0 | 1 | 0 | 1 | 0 | 1 | 0 | 1 | 1 | 1 | |
| R106 | 4 | 0 | 0 | 0 | 1 | 0 | 0 | 1 | 0 | 1 | 0 | 1 | 0 | 1 | |
| R107 | 6 | 0 | 0 | 0 | 0 | 0 | 0 | 1 | 0 | 1 | 0 | 0 | 1 | 0 | |
| R108 | 3 | 0 | 0 | 0 | 0 | 1 | 0 | 0 | 0 | 0 | 0 | 0 | 0 | 1 | |
| R109 | 3 | 0 | 0 | 1 | 1 | 1 | 0 | 0 | 0 | 0 | 0 | 0 | 1 | 1 | |
| R110 | 4 | 0 | 0 | 1 | 0 | 1 | 0 | 1 | 0 | 0 | 0 | 0 | 0 | 1 | |
| R111 | 4 | 0 | 0 | 0 | 1 | 0 | 0 | 0 | 0 | 0 | 0 | 0 | 1 | 1 | |
| R112 | 6 | 0 | 0 | 0 | 1 | 0 | 0 | 1 | 0 | 0 | 0 | 0 | 0 | 0 | |
| R113 | 4 | 0 | 0 | 0 | 1 | 1 | 0 | 1 | 0 | 0 | 0 | 0 | 1 | 0 | |
| R114 | 4 | 0 | 0 | 0 | 0 | 1 | 0 | 1 | 0 | 0 | 0 | 1 | 1 | 1 | |
| R115 | 3 | 0 | 0 | 0 | 0 | 1 | 0 | 1 | 0 | 0 | 0 | 1 | 0 | 0 | |
| R116 | 6 | 0 | 0 | 0 | 0 | 0 | 0 | 1 | 0 | 0 | 0 | 0 | 1 | 0 | |
| R117 | 5 | 0 | 0 | 0 | 0 | 0 | 0 | 1 | 0 | 1 | 0 | 0 | 0 | 1 | |
| R118 | 5 | 0 | 0 | 0 | 1 | 1 | 0 | 1 | 0 | 1 | 0 | 0 | 0 | 1 | |
| R119 | 3 | 0 | 0 | 0 | 0 | 1 | 0 | 1 | 0 | 0 | 0 | 1 | 0 | 1 | |
| R120 | 4 | 0 | 0 | 0 | 0 | 0 | 0 | 0 | 0 | 0 | 0 | 0 | 0 | 1 | |
| R121 | 1 | 0 | 0 | 0 | 0 | 1 | 0 | 1 | 0 | 0 | 0 | 1 | 0 | 1 | |
| R122 | 4 | 0 | 0 | 0 | 1 | 1 | 0 | 1 | 0 | 1 | 0 | 0 | 0 | 1 | |
| R123 | 4 | 0 | 0 | 0 | 1 | 1 | 0 | 1 | 0 | 0 | 0 | 0 | 0 | 1 | |
| R124 | 4 | 0 | 0 | 0 | 1 | 0 | 0 | 0 | 0 | 0 | 0 | 1 | 1 | 1 | |
| R125 | 1 | 0 | 0 | 0 | 0 | 0 | 0 | 1 | 0 | 0 | 0 | 0 | 0 | 1 | |
| R126 | 4 | 0 | 0 | 0 | 1 | 0 | 0 | 1 | 0 | 0 | 0 | 0 | 1 | 1 | |
| R127 | 4 | 0 | 0 | 0 | 0 | 0 | 0 | 1 | 0 | 0 | 0 | 1 | 0 | 1 | |
| R128 | 4 | 0 | 0 | 0 | 1 | 1 | 0 | 1 | 0 | 0 | 0 | 0 | 0 | 1 | |
| R129 | 3 | 0 | 0 | 0 | 1 | 1 | 0 | 1 | 0 | 1 | 0 | 1 | 1 | 1 | |
| R130 | 3 | 0 | 1 | 0 | 0 | 1 | 0 | 1 | 0 | 1 | 0 | 1 | 0 | 1 | |
| R131 | 3 | 0 | 1 | 0 | 0 | 1 | 0 | 1 | 0 | 0 | 0 | 0 | 0 | 1 | |
| R132 | 3 | 0 | 1 | 0 | 0 | 0 | 0 | 1 | 0 | 0 | 0 | 1 | 0 | 1 | |
| R133 | 1 | 0 | 0 | 0 | 1 | 0 | 0 | 1 | 0 | 0 | 0 | 0 | 1 | 1 | |
| R134 | 1 | 0 | 0 | 0 | 0 | 1 | 0 | 1 | 0 | 1 | 0 | 1 | 0 | 1 | |
| R135 | 3 | 0 | 0 | 0 | 0 | 1 | 0 | 1 | 0 | 0 | 0 | 0 | 0 | 1 | |
| R136 | 1 | 0 | 0 | 0 | 1 | 1 | 0 | 1 | 0 | 0 | 0 | 0 | 1 | 1 | |
| R137 | 4 | 0 | 0 | 0 | 0 | 1 | 0 | 1 | 0 | 0 | 0 | 0 | 0 | 1 | |
| R138 | 1 | 0 | 0 | 0 | 1 | 0 | 0 | 1 | 0 | 0 | 0 | 0 | 1 | 1 | |
| R139 | 5 | 0 | 0 | 0 | 0 | 0 | 0 | 1 | 0 | 0 | 0 | 0 | 0 | 1 | |
| R140 | 1 | 0 | 0 | 0 | 0 | 1 | 0 | 1 | 0 | 0 | 0 | 1 | 1 | 1 | |
| R141 | 8 | 0 | 0 | 0 | 1 | 0 | 0 | 1 | 0 | 1 | 0 | 0 | 0 | 0 | |
| R142 | 6 | 1 | 0 | 0 | 0 | 0 | 0 | 1 | 0 | 0 | 0 | 1 | 0 | 0 | |
| R143 | 4 | 0 | 1 | 0 | 0 | 1 | 0 | 1 | 0 | 1 | 0 | 1 | 0 | 0 | |
| R144 | 3 | 0 | 0 | 0 | 0 | 0 | 0 | 1 | 0 | 0 | 0 | 1 | 1 | 1 | |
| R145 | 4 | 0 | 1 | 0 | 0 | 1 | 0 | 0 | 0 | 0 | 0 | 1 | 1 | 1 | |
| R146 | 8 | 0 | 1 | 0 | 0 | 0 | 0 | 0 | 0 | 1 | 0 | 0 | 0 | 0 | |
| R147 | 5 | 0 | 0 | 1 | 0 | 1 | 0 | 1 | 0 | 0 | 0 | 1 | 1 | 0 | |
| R148 | 1 | 0 | 0 | 0 | 0 | 1 | 0 | 1 | 0 | 0 | 0 | 1 | 0 | 0 | |
| R149 | 1 | 0 | 1 | 0 | 0 | 1 | 0 | 1 | 0 | 0 | 0 | 0 | 0 | 1 | |
| R150 | 1 | 0 | 0 | 0 | 0 | 1 | 0 | 1 | 0 | 0 | 0 | 0 | 1 | 1 | |
| R151 | 5 | 0 | 0 | 0 | 0 | 1 | 0 | 1 | 0 | 0 | 0 | 0 | 0 | 1 | |
| R152 | 6 | 0 | 0 | 0 | 0 | 0 | 0 | 1 | 0 | 0 | 0 | 0 | 0 | 0 | |
| R153 | 3 | 0 | 0 | 0 | 0 | 1 | 0 | 0 | 0 | 0 | 0 | 0 | 1 | 0 | |
| R154 | 3 | 0 | 0 | 0 | 1 | 0 | 0 | 1 | 0 | 1 | 0 | 0 | 1 | 0 | |
| R155 | 5 | 0 | 0 | 0 | 0 | 0 | 0 | 1 | 0 | 1 | 0 | 0 | 1 | 1 | |
| R156 | 9 | 0 | 0 | 0 | 1 | 0 | 0 | 1 | 0 | 1 | 0 | 0 | 0 | 0 | |
| R157 | 9 | 0 | 0 | 1 | 1 | 0 | 0 | 1 | 0 | 1 | 0 | 0 | 0 | 0 | |
| R158 | 9 | 0 | 0 | 0 | 1 | 0 | 0 | 1 | 0 | 0 | 0 | 0 | 0 | 0 | |
| R159 | 9 | 0 | 0 | 1 | 0 | 0 | 0 | 1 | 0 | 1 | 0 | 0 | 0 | 0 | |
| R160 | 1 | 0 | 0 | 0 | 1 | 1 | 0 | 1 | 0 | 1 | 0 | 1 | 1 | 0 | |
| R161 | 3 | 0 | 1 | 0 | 0 | 1 | 0 | 0 | 0 | 0 | 0 | 0 | 1 | 0 | |
| R162 | 3 | 0 | 0 | 1 | 1 | 0 | 0 | 1 | 0 | 0 | 0 | 0 | 0 | 1 | |
| R163 | 3 | 0 | 0 | 1 | 0 | 1 | 0 | 1 | 0 | 0 | 0 | 1 | 1 | 0 | |
| R164 | 3 | 1 | 0 | 0 | 0 | 0 | 0 | 1 | 0 | 0 | 0 | 0 | 0 | 1 | |
| R165 | 5 | 1 | 0 | 0 | 1 | 0 | 0 | 1 | 0 | 0 | 0 | 0 | 0 | 1 | |
| R166 | 5 | 0 | 0 | 0 | 1 | 1 | 0 | 1 | 0 | 0 | 0 | 0 | 1 | 1 | |
| R167 | 5 | 0 | 0 | 0 | 0 | 1 | 0 | 1 | 0 | 0 | 0 | 1 | 0 | 1 | |
| R168 | 5 | 0 | 0 | 1 | 1 | 0 | 0 | 1 | 0 | 0 | 0 | 1 | 1 | 1 | |
| R169 | 5 | 0 | 0 | 1 | 0 | 0 | 0 | 1 | 0 | 0 | 0 | 1 | 0 | 1 | |
| R170 | 3 | 0 | 0 | 0 | 1 | 0 | 0 | 1 | 0 | 0 | 0 | 1 | 1 | 1 | |
| R171 | 3 | 0 | 0 | 0 | 0 | 1 | 0 | 1 | 0 | 0 | 0 | 0 | 0 | 1 | |
| R172 | 5 | 0 | 0 | 1 | 1 | 0 | 0 | 1 | 0 | 0 | 0 | 1 | 1 | 1 | |
| R173 | 4 | 0 | 0 | 0 | 0 | 1 | 0 | 1 | 0 | 0 | 0 | 1 | 0 | 1 | |
| R174 | 4 | 0 | 1 | 0 | 0 | 1 | 0 | 0 | 0 | 0 | 0 | 0 | 1 | 1 | |
| R175 | 3 | 0 | 0 | 0 | 1 | 0 | 0 | 1 | 0 | 0 | 0 | 1 | 1 | 1 | |
| R176 | 3 | 0 | 0 | 1 | 0 | 0 | 0 | 1 | 0 | 0 | 0 | 1 | 0 | 1 | |
| R177 | 3 | 0 | 0 | 1 | 1 | 0 | 0 | 1 | 0 | 0 | 0 | 1 | 0 | 1 | |
| R178 | 3 | 0 | 0 | 0 | 1 | 0 | 0 | 1 | 0 | 0 | 0 | 0 | 0 | 1 | |
| R179 | 3 | 0 | 0 | 0 | 0 | 1 | 0 | 1 | 0 | 0 | 0 | 0 | 0 | 1 | |
| R180 | 3 | 0 | 0 | 0 | 1 | 1 | 0 | 1 | 0 | 0 | 0 | 1 | 0 | 1 | |
| R181 | 4 | 0 | 0 | 0 | 0 | 0 | 0 | 1 | 0 | 0 | 0 | 0 | 0 | 1 | |
| R182 | 4 | 0 | 0 | 0 | 1 | 1 | 0 | 1 | 0 | 1 | 0 | 1 | 1 | 0 | |
| R183 | 7 | 0 | 0 | 0 | 0 | 0 | 0 | 1 | 0 | 0 | 0 | 0 | 0 | 0 | |
| R184 | 3 | 0 | 0 | 0 | 1 | 1 | 0 | 1 | 0 | 0 | 0 | 0 | 0 | 1 | |
| R185 | 4 | 1 | 0 | 0 | 0 | 1 | 0 | 1 | 0 | 0 | 0 | 0 | 1 | 0 | |
| R186 | 6 | 0 | 0 | 0 | 0 | 0 | 0 | 1 | 0 | 0 | 0 | 0 | 0 | 1 | |
| R187 | 4 | 0 | 0 | 0 | 0 | 0 | 0 | 1 | 0 | 0 | 0 | 1 | 1 | 0 | |
| R188 | 4 | 0 | 0 | 0 | 1 | 0 | 0 | 1 | 0 | 0 | 0 | 0 | 0 | 1 | |
| R189 | 4 | 0 | 0 | 0 | 0 | 1 | 0 | 1 | 0 | 0 | 0 | 1 | 1 | 0 | |
| R190 | 4 | 0 | 0 | 0 | 0 | 1 | 0 | 1 | 0 | 1 | 0 | 0 | 0 | 1 | |
| R191 | 3 | 0 | 0 | 0 | 0 | 0 | 0 | 1 | 1 | 0 | 0 | 1 | 1 | 1 | |
| R192 | 5 | 0 | 0 | 0 | 0 | 0 | 0 | 1 | 1 | 0 | 0 | 0 | 0 | 1 | |
| R193 | 3 | 0 | 1 | 0 | 0 | 1 | 0 | 1 | 0 | 1 | 0 | 0 | 0 | 1 | |
| R194 | 4 | 0 | 0 | 0 | 1 | 1 | 0 | 1 | 0 | 0 | 0 | 0 | 0 | 1 | |
| R195 | 3 | 0 | 0 | 0 | 1 | 0 | 0 | 1 | 0 | 1 | 0 | 0 | 0 | 1 | |
| R196 | 4 | 0 | 0 | 0 | 0 | 1 | 0 | 1 | 0 | 1 | 0 | 0 | 1 | 1 | |
| R197 | 4 | 0 | 0 | 0 | 0 | 1 | 0 | 1 | 0 | 0 | 0 | 0 | 0 | 1 | |
| R198 | 4 | 0 | 0 | 1 | 1 | 1 | 0 | 1 | 0 | 0 | 0 | 0 | 1 | 1 | |
| R199 | 5 | 0 | 1 | 0 | 0 | 1 | 0 | 1 | 0 | 0 | 0 | 0 | 0 | 1 | |
| R200 | 6 | 0 | 0 | 0 | 1 | 0 | 0 | 1 | 0 | 0 | 0 | 0 | 0 | 0 | |
| R201 | 4 | 0 | 0 | 1 | 0 | 1 | 0 | 1 | 0 | 1 | 0 | 1 | 0 | 0 | |
| R202 | 1 | 0 | 1 | 0 | 1 | 0 | 0 | 1 | 0 | 0 | 0 | 1 | 1 | 1 | |
| R203 | 4 | 0 | 1 | 0 | 0 | 1 | 0 | 0 | 0 | 1 | 0 | 0 | 0 | 1 | |
| R204 | 3 | 0 | 0 | 0 | 0 | 1 | 0 | 1 | 0 | 1 | 0 | 0 | 1 | 1 | |
| R205 | 4 | 0 | 0 | 1 | 1 | 1 | 0 | 1 | 0 | 0 | 0 | 0 | 0 | 0 | |
| R206 | 8 | 0 | 0 | 1 | 0 | 0 | 0 | 0 | 0 | 0 | 0 | 0 | 0 | 0 | |
| R207 | 4 | 0 | 0 | 0 | 0 | 1 | 0 | 1 | 0 | 1 | 0 | 0 | 1 | 0 | |
| R208 | 7 | 0 | 0 | 0 | 1 | 0 | 0 | 2 | 0 | 0 | 0 | 0 | 1 | 0 | |
| R209 | 1 | 0 | 1 | 0 | 0 | 1 | 0 | 0 | 0 | 0 | 0 | 0 | 0 | 1 | |
| R210 | 3 | 0 | 0 | 0 | 0 | 1 | 0 | 1 | 0 | 1 | 0 | 0 | 1 | 1 | |
| R211 | 3 | 0 | 0 | 1 | 1 | 1 | 0 | 1 | 0 | 1 | 0 | 1 | 0 | 0 | |
| R212 | 3 | 0 | 0 | 1 | 0 | 0 | 0 | 1 | 0 | 1 | 0 | 1 | 1 | 0 | |
| R213 | 3 | 0 | 0 | 1 | 1 | 0 | 0 | 1 | 0 | 0 | 0 | 1 | 0 | 1 | |
| R214 | 3 | 0 | 0 | 1 | 1 | 0 | 0 | 1 | 0 | 1 | 0 | 1 | 1 | 0 | |
| R215 | 3 | 0 | 0 | 1 | 0 | 1 | 0 | 1 | 0 | 1 | 0 | 1 | 1 | 1 | |
| R216 | 3 | 0 | 0 | 1 | 0 | 1 | 0 | 1 | 0 | 1 | 0 | 1 | 1 | 0 | |
| R217 | 3 | 0 | 1 | 1 | 0 | 1 | 0 | 1 | 0 | 0 | 0 | 1 | 1 | 1 | |
| R218 | 3 | 0 | 0 | 1 | 1 | 1 | 0 | 1 | 0 | 0 | 0 | 1 | 0 | 0 | |
| R219 | 3 | 0 | 0 | 0 | 0 | 1 | 0 | 1 | 0 | 1 | 0 | 1 | 0 | 1 | |
| R220 | 9 | 0 | 0 | 1 | 0 | 0 | 0 | 0 | 0 | 1 | 0 | 1 | 0 | 0 | |
| R221 | 1 | 0 | 0 | 0 | 0 | 1 | 0 | 1 | 0 | 0 | 0 | 0 | 0 | 0 | |
| R222 | 8 | 0 | 0 | 1 | 0 | 0 | 0 | 0 | 0 | 1 | 0 | 1 | 0 | 0 | |
|  |  |  |  |  |  |  |  |  |  |  |  |  |  |  | |

Table S4. Lists specific genetic markers related to stripe rust in wheat, aiding in the identification of resistance genes (*Yr*). Each row offers detailed information about the primers associated with a particular gene, including chromosomal locations, types of markers, sequences, annealing temperatures, and base-pair sizes for both the resistance (R allele) and susceptibility (S allele).

| **Primer Name** | **Chr location** | **Marker** | **Type** | **Pri. Sequence** | **Annealing (℃)** | **+**  **(bp)/R**  **allele** | **-(bp)/S allele** | **Reference** |
| --- | --- | --- | --- | --- | --- | --- | --- | --- |
| *Yr5* | 2BL | Yr5K | Gene specific | CTCACGCATTTGACCATATACAACT | 58 | 1200 | / | (Marchal et al. 2018) |
|  |  |  |  | TATTGCATAACATGGCCTCCAGT |  |  |  |  |
|  |  | Yr5_B F | SSR | GGGAACACTTCACGATCA | 53 | 909 | Null | (Marchal et al., 2018) |
|  |  | Yr5_B R |  | AATTCCTTCATGCCTTCC |  |  |  |  |
| *Yr9* | 1RS | IB-267 | STS | GCAAGTAAGCAGCTTGATTTAGC | 58 | 211 | / | (Mago et al., 2002) |
|  |  |  |  | AATGGATGTCCCGGTGAGTGG |  |  |  |  |
| *Yr 10* | 1BS | Xpsp3000 | SSR | GCAGACCTGTGTCATTGGTC | 54 | 237 | / | (Mukhtar et al., 2015) |
|  |  |  |  | GATATAGTGGCAGCAGGATACG |  |  |  |  |
|  | 4B | E51100 | SCAR | TCAAGGAGGTCAGTGACAG | 56 | 1085 | / | (Liu et al., 2014) |
|  |  |  |  | TCAGGGAGGTGTAGCCTAAT |  |  |  |  |
|  |  | Yr10K | Gene specific | TCAAAGACATCAAGAGCCGC | 51 | 543 | / | (Liu et al., 2014) |
|  |  |  |  | TGGCCTACATGAACTCTGGAT |  |  |  |  |
| *Yr15* | 1BS | uhw301 | Kinase I specific amplification primers | GGAGATAGAGCACATTACAGAC | 56 | 993 | / | (Klymiuk et al., 2018) |
|  |  |  |  | TTTCGCATCCCACCCATCTG |  |  |  |  |
|  |  | W_2F/R | Kinase II specific amplification primers | TGCACGCGGATATTAGGTAGG | 54 | 2015 | / | (Klymiuk et al., 2018) |
|  |  |  |  | TGATGAAGAGGACCAACGCA |  |  |  |  |
| *Yr17* | 2AS | URIC/LN2 | SSR | GGTCGCCCTGGCTTGCACCT | 64 | 285 | 275 | (Helguera et al., 2003) |
|  |  |  |  | TGCAGCTACAGCAGTATGTACACAAAA |  |  |  |  |
|  |  | VENTRIUP/LN2 | STS | AGGGGCTACTGACCAAGGCT |  | 259 bp | - | (Helguera et al., 2003) |
|  |  |  |  | TGCAGCTACAGCAGTATGTACACAAAA |  |  |  |  |
| *Yr18* |  | L34SPF | functional gene-specific | GGGAGCATTATTTTTTTCCATCATG | 58 | 751 | - | (Lagudah et al., 2009) |
|  |  | L34DINT13R2 |  | ACTTTCCTGAAAATAATACAAGCA |  |  |  |  |
|  |  | csLv34 | SSR | GTTGGTTAAGACTGGTGATGG | 55 | 150 | 250 | (Krattinger et al., 2009) |
|  |  |  |  | TGCTTGCTATTGCTGAATAGT |  |  |  |  |
| *Yr26* |  | CD77 | SSR | CGACGAAGCCGTTGTTAT | 55 | 480 | 620 | (Zhang et al., 2023) |
|  |  |  |  | TCAAGCAAAGACGAGAGGAT |  |  |  |  |
|  | 1B | STS-BQ74 | STS | TGGATGAACCAACGATAGT | 53 | 295Bp |  | (Dae Hee Han et al., 2015) |
|  |  |  |  | TGGGAAACACTTGACTGC |  |  |  |  |
| *Yr29* | 1BL | bac17R |  | CCCATGCTGACATGGCCACAT | 55 | 1700bp | 500 | (Rosewarne et al., 2005) |
|  |  |  |  | CTCTGCTCTTTAGTAGTTGCC |  |  |  |  |
| *Yr30* | 3BS | Xgwm533 | SSR | AAGGCGAATCAAACGGAATA |  | 115 |  | (Mawcha et al., 2022) |
|  |  |  |  | GTTGCTTTAGGGGAAAAGCC |  |  |  |  |
|  |  | Xgwm389 | SSR | ATCATGTCGATCTCCTTGACG |  | 115 |  | (Adhikari, Wallwork, & Goodwin, 2004) |
|  |  |  |  | TGCCATGCACATTAGCAGAT |  |  |  |  |
| *Yr36* | 6BS | WKS1_150F | Kinase-specific primers | ATGGAGCTCCCACGAAACAAAC | 57 | 857 | / | (Fu et al., 2009) |
|  |  | WKS1_620R |  | ACCTCCATGTTGCTCGCATTTGCT |  |  |  |  |
|  |  | Yr36E1a | gene specific | AAGGCAAAGGCAAAGTGG | 51 | 911 | / | (Gebreslasie et al., 2020) |
|  |  |  |  | TGATCTTTACCAAGCATTCG |  |  |  |  |
| *Yr48* | 5AL | BE495011 | EST-SSR | TGATTACTGTAGCTACCTCCTCCT | 56 | 236 | 228 | (Lowe et al*.,* 2011) |
|  |  |  |  | GGTGCAAGATGTGCCTGTAA |  |  |  |  |
|  |  | SNF-A2 | STS | TCCGTCTCCATCATTCAACA | 49 | 150 | 200 | (Lowe et al., 2011) |
|  |  |  |  | GTGTTGCGCAAGTTTGTGAC |  |  |  |  |
| *Yr65* | 1BS | gwm18 | SSR | TGGCGCCATGATTGCATTATCTTC | 53 | 182 | 188 | (Chen et al., 2014) |
|  |  |  |  | GGTTGCTGAAGAACCTTATTTAGG |  |  |  |  |
|  |  | gwm11 | SSR | GGATAGTCAGACAATTCTTGTG | 50 | 213 | 202 | (Bariana et al., 2007) |
|  |  |  |  | GTGAATTGTGTCTTGTATGCTTCC |  |  |  |  |
| *YrSP* |  | dp269 | STS | CTGCTGTCACCGCTCTCC | 55 | 198 | 209 | (Feng et al., 2015) |
|  |  |  |  | AGTCACACGCCCTACTCTCC |  |  |  |  |

Table S5. Different *Yr* genes associated with stripe rust resistance in wheat lines from Pakistan and China. The percentages represent the proportion of genotypes expressing *Yr* gene distribution in each population.

| *Yr* Genes | Historic lines Pakistan | % | Recent Cultivars Pakistan | % | Chuanyu wheat | % |
| --- | --- | --- | --- | --- | --- | --- |
| *Yr5* | 21 | 26% | 4 | 9% | 6 | 3% |
| *Yr9* | 25 | 31% | 8 | 17% | 26 | 12% |
| *Yr10* | 25 | 31% | 6 | 13% | 48 | 22% |
| *Yr15* | 66 | 83% | 29 | 62% | 92 | 41% |
| *Yr17* | 7 | 9% | 12 | 26% | 124 | 57% |
| *Yr18* | 19 | 24% | 17 | 36% | 1 | 0.4% |
| *Yr26* | 48 | 60% | 33 | 70% | 181 | 83% |
| *Yr29* | 0 | 0% | 9 | 19% | 37 | 17% |
| *Yr30* | 59 | 74% | 23 | 49% | 74 | 34% |
| *Yr36* | 39 | 49% | 13 | 28% | 0 | 0% |
| *Yr48* | 66 | 83% | 29 | 62% | 93 | 42% |
| *Yr65* | 50 | 63% | 28 | 60% | 164 | 38% |
| *YrSp* | 73 | 91% | 23 | 49% | 140 | 64% |

Table S6. Pedigree information of 80 historical wheat lines Pakistan. The pedigrees show the crossbreeding lineage that has influenced the molecular composition of each genotype. Notably, certain cultivars, including T-9, Shalakot-13, Faisalabad 85, Wdank-85, and SA-42, possess unknown pedigrees, signifying a need for documented crossbreeding data.

| **S.No** | **Accessions** | **Pedigree** |
| --- | --- | --- |
| G1 | T-9 | _ |
| G2 | T-11 | FORD/DONDEE ( I ) |
| G3 | Dirk | T9 × 8A |
| G4 | C-591 | HARD FEDERATION X 9D |
| G5 | C-228 | C 516 X C 591 |
| G6 | C-217 | Hard Federation x 9D |
| G7 | C-250 | C 209 × C 591 |
| G8 | C-273 | 21931-CHAPINGO53/ANDES SIB/3/Y50/4/C271/ PK937-17F-3K-2F |
| G9 | Khushal-69 | C271/WI(E)//SON64/ PK 146-12A-4A-0A |
| G10 | Chenab-70 | NAI60/CB151//S949/3/MEXIPAK |
| G11 | SA-42 | _ |
| G12 | Parula-73 | CNO67//SN64/KLRE/3/8156/ II23584-303M-0Y-11A-1A-1436-OPAK |
| G13 | Yacora | BLS/KHUSHAL |
| G14 | LU-26 | NAI60/CB151//S949/3/MEXIPAK/ PK6841-2A-2A-1A-0A |
| G15 | Punjab-76 | Nayab* (Fast neutran 600rads (Mutagensis) |
| G16 | Jauhar-78 | KVZ//BUHO//KAL/BB/ CM33027-F-15M-500Y-0M-76B-OY-OPAK |
| G17 | Pak 81 | FURY/KAL/BB CM37138-48Y-1M-5Y-1M-4V-5Y-0A-0PAK |
| G18 | Faisalabad 83 | BB/GLL/3/GTO/7C//BB/CN0/ CM32347-3M-1Y-1M-1Y-1K-0A-0PAK |
| G19 | Barani-83 | MAYA/MON//KVZ/TRM/ CM44083-N-3Y-1M-1Y-1M-1Y-OB |
| G20 | Faisalabad 85 |  |
| G21 | Wdank-85 | _ |
| G22 | Local White | Ciano"S" 67-8156 x Tobari-66-CNO 67/NOV-66/11-12300/LR6408156-PAN-76/ CM40577-6M-2Y-4M-4M-1Y-8M-1Y-0Y |
| G23 | Zardana | WL-711/CROW’S’ |
| G24 | Inqilab 91 | INIA66/A. DISTT//INIA66/3/GEN |
| G25 | Pasban 90 | Pavon Mutant-3 |
| G26 | Soghat-90 | JUP/BJYG//URES/ CM 67458-4Y-1M-3Y-1M-5Y-B |
| G27 | Bakhtawar 92 | TTR/JUN/ CM59123-3M-1Y-2M-1Y-2M-2Y-0M-0PAK |
| G28 | Kaghan 93 | WL 711//F3.71/TRM/ Pb 20371-20A-4A-0A-0K-0A |
| G29 | Shahkar 95 | SA 42 *2/3/CC/INIA4// BB/4/INIA/HD832/ Pb1352-B-4K-36A-0A |
| G30 | Punjab 96 | JUP/ALO"S"//KLT"S"/3/VEE"S"/ M79510-024Y-2M-05Y-01M-1Y-0B |
| G31 | Tatara | MEN’ SIB/MY48//4/14/3/YAYLA 305 |
| G32 | AZRI-96 | SA 42 *2/4CC/INIA//BB/3/ INIA/HD832/ Pb1352-B-4K-36A-0A |
| G33 | Punjab-96 | V-1562//CHRC`S'/HORK/3/KUFRA-I/4/CARP`S'/BJY`S' / Pb. 24883-B-1A.OA. |
| G34 | Kohistan 97 | BUC'S' / FCT'S' CM84663-7M-0Y-0M-7Y-0M |
| G35 | Chakwal-97 | SASONO KOMUGI/NORIN//BOB’S’ Pb18551-2B-1B-1B-2B-1B-0B |
| G36 | Darawar-97 | F12-71/COC//CNO79 CM76688-9Y-03M-02Y-2B-0Y |
| G37 | Dera-98 | Ures"s" / Bow/ CM78108-1M-02Y-02M-22Y-3B-0Y |
| G38 | Zarlashata | OPATA/BOW'S' CM 83398-2M-0Y-0M-5Y-0M |
| G39 | Magalla-99 | AU/UP301//GLL/Sx/3/PEW ‘S’/4/MAI ‘S’/MAY A ‘S’//PEW’S’/ CM.67245-C-2M-OY |
| G40 | Bahawalpur-2000 | CMH-77 A917/KPV-1600//RL-6010/6*SKA |
| G41 | Marvi-2000 | PASTOR/OPATA/ CM 110624-7M-020Y-010M-010SY-010M-0M-0Y |
| G42 | Amin-2000 | CHUM18/BAU/ CM91045 |
| G43 | Chenab-2000 | OPATA/RAYON//KAUZ CMBW 90Y3180-0TOPM-3Y-010M-010M-010Y-1M-015Y-0Y |
| G44 | Wafaq-01 | P20102//PIMA/SKA/3/TTR 'S' /BOW/ PB-23826-D-1A-1A-1T-1T-0T |
| G45 | Bahkhar-2002 | KHP/D31708//CM74A370/3/CIAN079/4/RL6043/*4NACPBD 795-23A-1A-0A. |
| G46 | AS-2002 | DWL5023/SNB//SNB CM84986-H-1M-3M-2B-0M |
| G47 | GA-2002 | KAUZ//ALTAR84/AOS/ CM 111633-6M-20Y-10M-10M-2Y-0M-0B. |
| G48 | Manthar-2003 | GEN#WHETON SWMI11508-LAP-1AP-1AP-4AP-1AP-5AP-0AP |
| G49 | KT-2000 | KAUZ/STAR |
| G50 | Pirsbak 2004 | VEE/TRAP#1//SOGHAT-90 |
| G51 | Bhittai | H-68XMAISXNORTENO |
| G52 | TD-1 | MUNIA/SHTO//AMSEL |
| G53 | Pirsabak 2005 | Cow"S"**/YACO//Cow"S" CRG873-5Y-010M-0Y |
| G54 | Rashkoh-2005 | BUC 'S'/4/TZPP//TRN46/CN067/3/PRT / FLAKE 56744-7Y-2Y-1M-0M |
| G55 | Moomal 2002 | CHIL/2*STAR/4/BOW/CROW//BUC/PVN/3/…. |
| G56 | Sehar -2006 | LU26/HD2179//2*INQ-91 |
| G57 | Shafaq -2006 | CHIL / ALD // PVN / Yecora-70 |
| G58 | Saussi | PTS/3/TOB/LFN//BB/4/BB/HD-832-5//ON/5/GV/ALD’S’//HPO’S’ BR-3385-3B-1B-OB |
| G59 | Fareed-06 | HD-2329 PAU-ACC-3079 |
| G60 | SKD-1 | ULC/PVN//TAN/3/BUC |
| G61 | Khirman | ATTILA/3/HUI/CARC//CHEN/CHTO/4/ATTILACMW90M48601-0T-TOPY-16M-1Y-010M-010Y-1M-015Y-0Y |
| G62 | Chakwal-50 | LUAN/Koh-97 |
| G63 | Lasani-08 | SPARROW/INIA//V.7394/WI-711/3/BAU’S’ BR.2974-2B-1B-9B-0B |
| G64 | Meraj-08 | PBW-65/2*PASTOR |
| G65 | Faisalabad-08 | PFAU/JUN//KAUZ / CM96818-1-0Y-0M-0B-2Y-2Y-0M |
| G66 | Bathoor-08 | ATTILA CM 85836-4Y-0M-0Y-14M-0Y-5M-0Y-1SJ-0Y- |
| G67 | Gomal-08 | JUP/ALD’S’//KLT’S’/3/VEE’S’/6/BEZ//0APTOB/8156/4/ON/3/6*TH/KF//6* LEE/KF/5 / ICW91-0321-2AP-0TS-1AP-2AP-0L-0AP |
| G68 | Hashim-08 | INQALAB 91*2/TUKURU CGSS99B00015F-099Y-099M-099Y-099M-29Y-0B-0ID |
| G69 | NARC-2009 | PFAU/SERI//BOW CM85295-101TOPY-2M-0Y-0M-3Y-0M-0SY |
| G70 | BARS-2009 | FRET2 / CGSS96Y00146T-099B-099Y-099B-16Y-0B-0SY |
| G71 | NIFA-Barsat10 | VEE//5 'S'/SARA//SOGHAT90 |
| G72 | NIA-Amber | Cham4//URES/BOW 'S' |
| G73 | NIA-Sunehri | GEN*2//BUC/FILK/3/BUCHIN |
| G74 | Janbaz | INQILAB 91*2/TUKURU    CGSS99B00015F-099Y-099M-099Y-31Y- OB |
| G75 | Atta Habib | ALTAR84/AE.SQUARROSA.(219)01//SERIBW91Y008925-8Y-11KBY-2KBY-010M-9Y-3M-0Y-05Y |
| G76 | Punjab-2011 | CHENAB2000/INQ-91 |
| G77 | Millat-2011 | LFN/1158.57//PRL/3/HAHN/4/KAUZ/5/KAUZ/ CMB89Y1044-0T0PM-8Y-010M-020B-0NPL-010Y-3M-015Y-0Y |
| G78 | Hamal-Faqir | PB96/87094/MH97 PB30332-0A-0A-0A-9A-27A |
| G79 | Galaxy-2013 | TRACHA’S//CMH76-252/PVN’S |
| G80 | Shalakot-13 |  |

Table S7. Pedigree information recent cultivars from Pakistan. Each entry includes a variety of names and detailed breeding information (Pedigree) that outlines the genetic lineage of each cultivar. Notably, certain cultivars, including D30, D31 …... and D47 possess unknown pedigrees, signifying a deficiency in documented crossbreeding data.

| **S.No#** | **Varieties** | **Pedigree** |
| --- | --- | --- |
| D1 | Pakhtunkhwa-15 | WBLLI*2/4/YACO/PBW65/3/KAUZ*2/TRAP//KAUZ/ CGSS01Y00054T-099M-099Y-099M-099M-20Y-0B |
| D2 | IBGE-Ghaneemat | CROC-1/AE.SQUARROSA(205)//KAU2/3/ATTILA/ CMSS93Y01031S-13Y-5KBY-010M-010Y-5M-0KBY-0M-9KBY- … |
| D3 | NIFA-Insaf | Tatara/Inqilab-91 |
| D4 | Zincol | CROC_AE.SQUARROSA(210)//INQILAB-91*2/KUKUNA /3/ PBW343* 2/ KUKUNA /CMSA06M00195-099Y-099Y-099M-9M-0Y-7B-0Y-0ID |
| D5 | Boroloug-16 | RFUG99RE 09/10 CIMMYT Selection#08 (2009-10) /Ptss02b00015S-0Y-0B-0Y-1Y-0M-0SY-0ID |
| D6 | NIFA-Aman | PRL/2*PASTOR//PBW343*2/KUKUNA/3/ROLY07 /29CMSS04B00025T-0TOPY-09922TM-099Y-8WGY-0B |
| D7 | Ihsan-16 | SOKOLLl |
| D8 | Khaista-17 | KAUZ//ALTAR 84/AOS/3/MILAN/KAUZ/4/HUITES/7/ CAL/NH//H567.71/3/SERI/4/CAL/ NH//H567.71 /5/2*KAUZ/6/PASTOR /CMSS05B00581S-099Y-099M-099Y-099ZTM-2WGY-0B |
| D9 | Israr Shaheed | ESDA/ / ALTAR 84 / AE.AQUARROSA (211) /3/ ESDA/4/ / CMSS96M01591S-050M-040Y-0100M-020Y-3M-0Y |
| D10 | Shahid-17 | BYJ/COC/PRL/BOW/3/URES/JUN/KAUZ/ CMSS96Y02566S-040Y-020M-050SY-020SY-15M-0Y |
| D11 | Kohat-17 | SOKOLL/WBLLI/ /PTSS02Y00021S-0Y-030ZTM-040SY-040M-5Y-0M-0SY |
| D12 | Anaj-2017 | BABAX/LR43//BABAX/6/MOR/VEE#5//DUCULA/3/DUCULA/4/MILAN/5/BAU/MILAN/7/SKAUZ/BAV92 |
| D13 | Barani-17 | SOKOLL/EXCALIBER / CNSA04Y00612S-28ZTPOY-010M-010SY-4M-02Y-0B |
| D14 | Bhakkar Star | CHEN/AEGILOPS SQUARROSA(TAUS)//BCN/3/AV92/ CMSA02Y00104S-040P0Y-040ZTM-040SY-040M-10ZTY-02M |
| D15 | Markaz-2019 | MUTUS*2/HARIL#1 |
| D16 | Faheem-19 | MILAN/KAUZ//PRINIA/3/BAV92/4/2*SOKOL |
| D17 | Ghazi-2019 | WBLL*2/4/4YACO/PBW.65/3/KAUZ*2/TRAP//KAUZ/5/….. CMSS05B00061S-099Y-099M-099Y-099ZTM-5WGY-0B |
| D18 | NIA-Zarkhaz | KAUZ/Gen |
| D19 | NIA-Shaheen | Kiran/Khirman/Bhittai |
| D20 | Gulzar-19 | VORB/3/T.DICOCCON9194625/AE.SQUARROSA(372)//3*PASTOR |
| D21 | Pirsbak-19 | NAC/THAC//3*PVN/3/MIRLO/BUC/4/2*PASTOR/5/KACHU/6/KACHU |
| D22 | Aghaz | Croc.1/A.E SQUARRAOSA(205)11KAUZ/PASTOR CMSS96-M1560S-0S-0M-040Y-0100M-020Y-41M-0Y/ |
| D23 | AZRC DK | BECARD/KACHU/ CMSS06B00169S-0Y-099ZTM-099Y-099M-28WGY-0B |
| D24 | Abbasen-21 | SALEEM-200 X PS-05/ CCSS10-11S-OCC-OK-5006CC1-OK-7016CC1-OK-9005CC1 |
| D25 | NARC Super | MUTUS*2/HARIL#1 |
| D26 | Subhani | KACHU/SAUAL*2/5/SERI.1B//KAUZ/HEVO/3/AMAD*2/4/KIRITATI /CMSS10B010307-099TOPY-099M-099NJ-WGY-0B |
| D27 | Dilkash | WBLL*2/4/SNI/TRAP”1/3/KAUZ*2/TRAP//KAUZ/5/PB96/Watan |
| D28 | Nawab-21 | 1G094.7.1.12/2*QUAIU#/3/VIUAJUAREZ F22009/SPLALA//.. |
| D29 | Saddiq-21 | QUAIU/5/UP2338*2/SHAMA/3/MILAN/KAUZ/CHIL/… |
| D30 | Pirs bak-15 | - |
| D31 | Ujala-15 | - |
| D32 | Sindhu-16 | - |
| D33 | Gold-16 | - |
| D34 | Johar16 | - |
| D35 | Fatehjang16 | - |
| D36 | NN-Gandum1 | - |
| D37 | Wardan 17 | - |
| D38 | Pasina17 | - |
| D39 | Famhr-e-Bhakkar- | |
| D40 | NIFA-Awaz | - |
| D41 | Akber 19 | - |
| D42 | Khosha | - |
| D43 | Umeed-e-Khas | - |
| D44 | Pirsabak21 | - |
| D45 | Zargoon-21 | - |
| D46 | Swabi 1 | - |
| D47 | Nishan | - |

Table S8. List of 222 genotypes of Chuanyu wheat, including certificate numbers for varieties. Sixteen of these entries are classified as approved varieties; each assigned a specific certificate number, while the remaining entries consist of advanced breeding lines.

| **S.No** | **Accessions** | **Remarks** |
| --- | --- | --- |
| R1 | Chuanyu20 | certificated by China (Sichuan No.2007001) |
| R2 | Chuanyu21 | certificated by Sichuan Province(Sichuan No.2007007) |
| R3 | Chuanyu23 | certificated by Sichuan Province (Sichuan No.2008003) |
| R4 | Chuanyu24 | certificated by Sichuan Province (Sichuan No.2009005) |
| R5 | Chuanyu25 | certificated by Sichuan Province (Sichuan No.2015012) |
| R6 | Chuanyu26 | certificated by Sichuan Province (Sichuan No.2016009) |
| R7 | Chuanyu27 | certificated by Sichuan Province(Sichuan No.2017004) |
| R8 | Chuanyu29 | certificated by Sichuan Province(Sichuan No.20180006) |
| R9 | Chuanyu31 | certificated by Sichuan Province(Sichuan No.20190008) |
| R10 | Chuanyu32 | certificated by Sichuan Province(Sichuan No.20210001) |
| R11 | Chuanyu33（present） |  |
| R12 | Chuanyu33（primary） | certificated by Chongqing City(Chongqing No.20220005) |
| R13 | Chuanyu34-2（17G179) |  |
| R14 | Chuanyu34-1（17G178) |  |
| R15 | Chuanyu35-3 | certificated by Sichuan Province (Sichuan No.20200017) |
| R16 | Chuanyu37 | certificated by Guizhou Province(Guizhou No.20220002) |
| R17 | Chuanyu38 |  |
| R18 | Chuanyu40 | certificated by Chongqing City(Chongqing No.20240004) |
| R19 | Chuanyu42 | certificated by Sichuan Province(Sichuan No.20200002) |
| R20 | Chuanyu44-2（XianChuanyu61） |  |
| R21 | Chuanyu44-3 |  |
| R22 | Chuanyu45-3 |  |
| R23 | Yu mai9hao |  |
| R24 | Chuanyu46-1 |  |
| R25 | Chuanyu46-2 |  |
| R26 | Chuanyu43-2 |  |
| R27 | Chuanyu47 |  |
| R28 | Chuanyu48Bai li |  |
| R29 | Chuanyu48（YuanYu mai7hao） |  |
| R30 | Yu mai6hao | certificated by Sichuan Province(Sichuan No.20220004) |
| R31 | Chuanyu63（YuanYu mai6hao） |  |
| R32 | Chuanyu49 |  |
| R33 | Chuanyu51 |  |
| R34 | Chuanyu52 |  |
| R35 | Chuanyu53 |  |
| R36 | XianYu mai7hao（YuanChuanyu101） |  |
| R37 | 21A1(925short//00062/Mian nong4hao） |  |
| R38 | Chuanyu62 |  |
| R39 | Chuanyu60 |  |
| R40 | Chuanyu65 |  |
| R41 | Chuanyu66 |  |
| R42 | 41058 ×（Mo444/90-7//085/10927） |  |
| R43 | (G214/41058)×(Ji mai20/G323) |  |
| R44 | 925short×（41058/4/R59/3/Mo457/8619-10//Yibin bai maizi） |  |
| R45 | (G214/41058)×Yibin bai maizi |  |
| R46 | Chuanyu23/15C2(38051/3/Chuanyu112/Gui nong20/10927) |  |
| R47 | 15G148 × 16G1097 |  |
| R48 | 13G421×P89074-1-1 |  |
| R49 | 925short/31520 |  |
| R50 | Heng guan35 ×G435(G214/41058) |  |
| R51 | 925shortTao dai zi jiao |  |
| R52 | 14-23087(34756/Yan nong19) × 15G312(92R/R88/3/92R139/Chuanyu12//30389) |  |
| R53 | 14-23087(34756/Yan nong19) × 15G312(92R/R88/3/92R139/Chuanyu12//30389) |  |
| R54 | 34756//Xiao yan166/00062 |  |
| R55 | 30389/Zhou mai22 |  |
| R56 | (G214/41058) ×G18(Jin ding-1/41058) |  |
| R57 | (G214/41058)×(Jin ding-1/41058) |  |
| R58 | 18-33324Jiaozhi: 925short×(SW8688/YunFan58254) |  |
| R59 | Heng guan35 ×G435(G214/41058) |  |
| R60 | qin830×12B6(G211/30389) |  |
| R61 | 925short × 15G392(Zhong you95-7/03Pin fu(1)//Fu nong/G219) |  |
| R62 | WAMI283*Chuanyu27 |  |
| R63 | 925short × 15F4A(92R-3/R88/3/92R139/Chuanyu12//30389) |  |
| R64 | 16G9(38051/H291) × 16G841(925short//00062/Mian nong4hao) |  |
| R65 | 16G9(38051/H291) × 16G841(925short//00062/Mian nong4hao) |  |
| R66 | 16G9(38051/H291) × 16G841(925short//00062/Mian nong4hao) |  |
| R67 | 17G5(38051/H291)*17-19680(34756/Shi02-6207) |  |
| R68 | WAMI34*17G21(41058/3/Mo444/90-7//085/10927)) |  |
| R69 | SW8688/Yunfan88208/Nei2889 |  |
| R70 | SW8688/Yunfan88208/Nei2889 |  |
| R71 | 925short/12G65 |  |
| R72 | 34756//34756/Yan nong19 |  |
| R73 | qinBen tichun 16G841（925short//00062/Mian nong4hao） |  |
| R74 | 18-11302-3: WAMI80/17C1(G34/30389) |  |
| R75 | 14-25582(38051/Chuanyu12-4) × 15G38(Chuanyu12/925short) |  |
| R76 | 14-25582(38051/Chuanyu12-4) × 15G38(Chuanyu12/925short) |  |
| R77 | 14G226(Shi02-6207/3/Zhong zhi3586/50669//98(0)2/11020) × Ya anZao |  |
| R78 | (G214/41058) ×qin420(qinR138Lan) |  |
| R79 | 14G226(Shi02-6207/3/Zhong zhi3586/50669//98(0)2/11020) × Ya anZao |  |
| R80 | YunHun4-9 |  |
| R81 | Zhong zhi/Zhang wu × 14Sheng2(10Jian236,Jian38/99116//Chuan mai42) |  |
| R82 | Zhong zhi/Zhang wu × 15G32(925short/G323) |  |
| R83 | 21-Chuanyu25Zao |  |
| R84 | 21-Chuanyu25Chi |  |
| R85 | Chuanyu44 |  |
| R86 | Chuanyu46 |  |
| R87 | Chuanyu23/15E8(34756///Xiao yan166/00062） |  |
| R88 | Heng guan35 ×G435(G214/41058) |  |
| R89 | (G214/41058) ×Chuanyu20hao |  |
| R90 | 925short/31520 |  |
| R91 | 13-19538(G214/31520)/13Sheng2(Chuanzhong125,Chuan mai42/Chuan nong16) |  |
| R92 | 18-33324Jiaozhi: 925short×(SW8688/YunFan58254) |  |
| R93 | (G214/41058) ×G18(Jin ding-1/41058) |  |
| R94 | 14G226(Shi02-6207/G214)/Chuanyu23 |  |
| R95 | 41058 ×（Mo444/90-7//085/10927） |  |
| R96 | S13/Chuanyu20 |  |
| R97 | 925short × 15G470(G214/3/H163//45661/Yun66518) |  |
| R98 | 14G41(38051/G323)/14Sheng5(11P6-8,99-1572/SW8688//01-3570) |  |
| R99 | 925short×G372(SA9331/Ge lan ni//Chuanyu16) |  |
| R100 | 925short×G372(SA9331/Ge lan ni//Chuanyu16) |  |
| R101 | 13-19538(G214/31520)/13Sheng2(Chuanzhong125,Chuan mai42/Chuan nong16) |  |
| R102 | 925short×G32(38051/41058) |  |
| R103 | G34/15G129(30389/3/G394//30024/NE7 |  |
| R104 | (G214/41058)×(Ji mai20/G323) |  |
| R105 | 925short×15610 |  |
| R106 | 925short×15610 |  |
| R107 | 925short/G421 |  |
| R108 | 17G21(41058/3/Mo444/90-7//085/10927)*17G170(G211/30389) |  |
| R109 | 17G21(41058/3/Mo444/90-7//085/10927)*17-21840(Chuan mai42Kang-6/13B2(G219/3/ve/35050//G495/qin2194)) |  |
| R110 | 17G5(38051/H291)*17-8361(qin16G92) |  |
| R111 | 17C9(Jì mài)20/46725)*WAMI-283 |  |
| R112 | WAMI34/3/925short//Zhong zhi3568/50669/4/ 925short/04G368(Zhong zhi3568/50669) |  |
| R113 | 14G226 × 15G473(SA9331/Ge lan ni//Chuanyu16) |  |
| R114 | 13-19538(G214/31520)/14G6(Jin ding-1/41058) |  |
| R115 | 04G368×Wen mai6hao |  |
| R116 | WAMI34//G34/30389/3/925short//00062/Mian nong4hao |  |
| R117 | WAMI34/3/G219/Ve35050//99G495/qin21947/4/ WAMI34 |  |
| R118 | 34756//34756/Yan nong19/3/ Shu mai133 |  |
| R119 | Chuanyu23/17C5(Zhong you95-7/03Pin fu(1)//Fu nong/G219) |  |
| R120 | 15G148 × Chuanyu20 |  |
| R121 | 15G148 × 16G1102 |  |
| R122 | 16G376(Chuan mai42/3/Zhong zhi3586/50669//98(0)2/11020) × 16G1102 |  |
| R123 | 16G1(R59//Zheng9023/H435) × Fan xuan4 |  |
| R124 | 16G9(38051/H291) × 16G29(G214/3/H163//45561/Yun66518) |  |
| R125 | 16G9(38051/H291) × 16G881(G214/4/Chuanyu12//Gui nong20/10927/3/41058) |  |
| R126 | 16G29(G214/3/H163//45561/Yun66518) × 16G34(34756//34756/Zhong youXuan3) |  |
| R127 | 16G29(G214/3/H163//45561/Yun66518) × 16G34(34756//34756/Zhong youXuan3) |  |
| R128 | 16G29(G214/3/H163//45561/Yun66518) × 16G881(G214/4/Chuanyu12//Gui nong20/10927/3/41058) |  |
| R129 | 16G29(G214/3/H163//45561/Yun66518) × 16G1102 |  |
| R130 | 16G29(G214/3/H163//45561/Yun66518) × 16G1102 |  |
| R131 | 16G29(G214/3/H163//45561/Yun66518) × 16G1102 |  |
| R132 | G34 × 16G981(R57//Xiao yan54/G323) |  |
| R133 | 925short/17-19680(34756/Shi02-6207) |  |
| R134 | 1346(14G217/14G550)×14Sheng6(Chuan11131,01-3570/R138) |  |
| R135 | WAMI34//34756/Shi02-6207/3/Ji tian16c3324 |  |
| R136 | 12C8m1-1/Guo qu4 |  |
| R137 | 16G186(G219/Ve35050//99G495/qin21947）/17E13(925short/15610) |  |
| R138 | 17G21(41058/3/Mo444/90-7//085/10927)*17G170(G211/30389) |  |
| R139 | WAMI34//34756/Shi02-6207/3/ G214/31520 |  |
| R140 | 17G21(41058/3/Mo444/90-7//085/10927)*17G170(G211/30389) |  |
| R141 | 14Sheng2 × 16G1102 |  |
| R142 | WAMI34/16G184(G219//Zheng9023/Zhong you95-7) |  |
| R143 | Chuanyu23/17-1209((2015M8323),Zhang sui) |  |
| R144 | Chuanyu23/17-1209((2015M8323),Zhang sui) |  |
| R145 | 925short/17D3(41058/3/Yun1752-5//H35/Lang9247) |  |
| R146 | 925short × 15B6(Chuanyu12/09G166) |  |
| R147 | 925short × 15G392(Zhong you95-7/03Pin fu(1)//Fu nong/G219) |  |
| R148 | G214×(Chuanyu12//Gui nong20/10927/3/41058) |  |
| R149 | Xiao hei mai-2 |  |
| R150 | CY12/16F689 F9 Bai li |  |
| R151 | WAMI/CY27 |  |
| R152 | 17C9(Ji mai20/46725)/WAMI-8 |  |
| R153 | 14-23087(34756/Yan nong19) × 15G312(92R/R88/3/92R139/Chuanyu12//30389) |  |
| R154 | 14-23087(34756/Yan nong19) × 15G312(92R/R88/3/92R139/Chuanyu12//30389) |  |
| R155 | 925short/15G392(Zhong you95-7/03Pin fu（1）//Fu nong/G219 |  |
| R156 | CY12Tubian ti shao zao |  |
| R157 | CY12Tubian ti shao zao |  |
| R158 | CY12Tubian ti shao zao |  |
| R159 | CY12Tubian ti shao zao |  |
| R160 | 13-19538(G214/31520)/13Sheng2(Chuanzhong125,Chuan mai42/Chuan nong16) |  |
| R161 | WAMI34*17G21(41058/3/Mo444/90-7//085/10927)) |  |
| R162 | 14G226(Shi02-6207/3/Zhong zhi3586/50669//98(0)2/11020) × 14Sheng2 |  |
| R163 | 14G226 × 15G487(GWu pai2-2-2) |  |
| R164 | 14G226 × 15G473(SA9331/Ge lan ni//Chuanyu16) |  |
| R165 | Chuanyu20 × Plot225 |  |
| R166 | 18-11288-1: WAMI80/17F6(Chuanyu25/Chuan mai107) |  |
| R167 | 18-11288-1: WAMI80/17F6(Chuanyu25/Chuan mai107) |  |
| R168 | WAMI34//G18/Chuan mai107/3/ 41058/3/Mo444/90-7//085/10927 |  |
| R169 | 16G184(G219//Zheng9023/Zhong you95-7) × Plot173 |  |
| R170 | 15G148 × 16G1102 |  |
| R171 | 16G9(38051/H291) × 16G881(G214/4/Chuanyu12//Gui nong20/10927/3/41058) |  |
| R172 | 16G29(G214/3/H163//45561/Yun66518) × 16E2(34756/Chuanyu20) |  |
| R173 | 14G226×Chuan mai42-6 |  |
| R174 | Xiao yan54/G323-2-4 |  |
| R175 | 925short//sw8688/Yunfan58254（Chuanyu16） |  |
| R176 | (G214/41058) ×Chuanyu20hao |  |
| R177 | (G214/41058)×Heng guan35 |  |
| R178 | G214/00062//12G65 |  |
| R179 | (G214/41058)×qin744(03G211/41058) |  |
| R180 | 38051/4/00062/3/Mo444/90-7//085/10927 |  |
| R181 | 16G29(G214/3/H163//45561/Yun66518) × 16G1102 |  |
| R182 | 16G29(G214/3/H163//45561/Yun66518) × 2D2157 |  |
| R183 | 16-1564(Chuanyu23//Chuanyu12/925short) × Luohe88076 |  |
| R184 | 17G21(41058/3/Mo444/90-7//085/10927)*17G5(38051/H291) |  |
| R185 | Chuanyu20 × Plot225 |  |
| R186 | 13-19538(G214/31520)/13Sheng2(Chuanzhong125,Chuan mai42/Chuan nong16) |  |
| R187 | 13-19538(G214/31520)/14G6(Jin ding-1/41058) |  |
| R188 | (G214/41058)×R57(Chuan nong17) |  |
| R189 | 34756×（34756//00062/Mian yang26） |  |
| R190 | 13-19538(G214/31520)/13Sheng2(Chuanzhong125,Chuan mai42/Chuan nong16) |  |
| R191 | WAMI34/3/925short//Zhong zhi3568/50669/4/ 925short/04G368(Zhong zhi3568/50669) |  |
| R192 | WAMI34/Chuanyu27// Chuanyu27 |  |
| R193 | 17G5(38051/H291)*15Guo quShi14 |  |
| R194 | 17G21(41058/3/Mo444/90-7//085/10927)*17B6(925short//00062/Mian nong4hao) |  |
| R195 | 17G21(41058/3/Mo444/90-7//085/10927)*17-21840(Chuan mai42Kang-6/13B2(G219/3/ve/35050//G495/qin2194)) |  |
| R196 | 14G37(38051/G323)/14G109(925short//H291/Zhong you95-7) |  |
| R197 | Chuanyu23/15B2(04G214/41058) |  |
| R198 | 30389/Chuanyu19 |  |
| R199 | 14G41(38051/G323)/14Sheng5(11P6-8,99-1572/SW8688//01-3570) |  |
| R200 | WAMI285//G214/41058 |  |
| R201 | WAMI34/3/925short//Zhong zhi3568/50669/4/925short/04G368(Zhong zhi3568/50669) |  |
| R202 | WAMI34//34756/Shi02-6207/3/ 34756/2339 |  |
| R203 | Chuanyu20 × Plot225 |  |
| R204 | 13-19538(G214/31520)/14G6(Jin ding-1/41058) |  |
| R205 | G34 × 16G337(Chuan mai42Kang/3/92R139/Chuanyu12//30389) |  |
| R206 | 41058 ×（Mo444/90-7//085/10927） |  |
| R207 | (G214/41058)×(Jin ding-1/41058) |  |
| R208 | Chuanyu38 |  |
| R209 | 17G5(38051/H291)/17-19680(34756/Shi02-6207) |  |
| R210 | （925short × 12D7(G214/41058)）*19A13 |  |
| R211 | （925short × 12D7(G214/41058)）*19A13 |  |
| R212 | （925short × 12D7(G214/41058)）*19A13 |  |
| R213 | （925short × 12D7(G214/41058)）*19A13 |  |
| R214 | （925short × 12D7(G214/41058)）*19A13 |  |
| R215 | （925short × 12D7(G214/41058)）*19A13 |  |
| R216 | （925short × 12D7(G214/41058)）*19A13 |  |
| R217 | （925short × 12D7(G214/41058)）*19A13 |  |
| R218 | Chuanyu47-2 |  |
| R219 | Chuanyu47-4 |  |
| R220 | CY12 |  |
| R 221 | Hussar |  |
| R222 | YZ1 |  |

**References**

1. Adhikari, T. B., Wallwork, H., & Goodwin, S. B. (2004). Microsatellite markers linked to the *Stb2* and *Stb3e* gnes for resistance to *Septoria Tritici* blotch in wheat. *Crop Science*, 44(4), 1403–1411. doi:10.2135/cropsci2004.1403
2. Bariana, H. S., Brown, G. N., Bansal, U. K., Miah, H., Standen, G. E., & Lu, M. (2007). Breeding triple rust resistant wheat cultivars for Australia using conventional and marker-assisted selection technologies. *Australian Journal of Agricultural Research*, 58(6), 576–587. doi:10.1071/ar07124
3. Chen, W., Wellings, C., Chen, X., Kang, Z., & Liu, T. (2014). Wheat stripe (yellow) rust caused by *Puccinia striiformisf*. sp.*tritici.* *Molecular Plant Pathology,* 15(5), 433–446. doi:10.1111/mpp.12116
4. Dae Hee Han, Wang, Q., Chen, X. R., Zeng, Q. L., Wu, J., Xue, W. B., et al. (2015). Emerging *Yr26*-virulent races of *Puccinia striiformis* f. *tritici* are threatening wheat production in the Sichuan Basin, China. *Plant Disease*, 99(6), 754–760. doi:10.1094/pdis-08-14-0865-re
5. Feng, J. C., Wang, M., Chen, X. R., See, D. R., Zheng, Y. H., Chao, S., & Wan, A. (2015). Molecular mapping of *YrSP* and its relationship with other genes for stripe rust resistance in wheat chromosome 2BL. *Phytopathology*, 105(9), 1206–1213. doi:10.1094/phyto-03-15-0060-r
6. Fu, D., Uauy, C., Assaf Distelfeld, Blechl, A. E., Epstein, L., Chen, X., et al. (2009). A Kinase-START gene confers temperature-dependent resistance to wheat stripe rust. *Science*, *323*(5919), 1357–1360. doi:10.1126/science.1166289
7. Gebreslasie, Z. S., Huang, S., Zhan, G., Badebo, A., Zeng, Q., Wu, J., et al. (2020). Stripe rust resistance genes in a set of Ethiopian bread wheat cultivars and breeding lines. *Euphytica*, *216*(2), 1–14 .doi:10.1007/s10681-019-2541-z
8. Helguera, M., Khan, I. A., Kolmer, J., Lijavetzky, D., Zhong‐qi, L., & Dubcovsky, J. (2003). PCR assays for the *Lr37‐Yr17‐Sr38* cluster of rust resistance genes and their use to develop isogenic hard red spring wheat lines. *Crop Science*, *43*(5), 1839–1847. doi:10.2135/cropsci2003.1839
9. Klymiuk, V., Yaniv, E., Huang, L., Raats, D., Fatiukha, A., Chen, S., et al. (2018). Cloning of the wheat *Yr15* resistance gene sheds light on the plant tandem kinase-pseudokinase family. *Nature Communications*, *9*(1), 1–12. doi.:10.1038/s41467-018-06138-9
10. Krattinger, S. G., Lagudah, E. S., Spielmeyer, W., Singh, R. P., Huerta-Espino, J., McFadden, H., et al. (2009). A putative ABC transporter confers durable resistance to multiple fungal pathogens in wheat. *Science*, 323(5919), 1360–1363. doi:10.1126/science.1166453
11. Lagudah, E. S., Krattinger, S. G., Herrera-Foessel, S., Singh, R. P., Huerta-Espino, J., Spielmeyer, W., et al. (2009). Gene-specific markers for the wheat gene *Lr34/Yr18/Pm38* which confers resistance to multiple fungal pathogens. *Theoretical and Applied Genetics*, *119*(5), 889–898. doi:10.1007/s00122-009-1097-z
12. Liu, W., Frick, M., Huel, R., Nykiforuk, C. L., Wang, X., Gaudet, D. A., et al. (2014). The stripe rust resistance gene *Yr10* encodes an evolutionary-conserved and unique CC–NBS–LRR sequence in wheat. *Molecular Plant*, *7*(12), 1740–1755. doi:10.1093/mp/ssu112
13. Lowe, I., Jankuloski, L., Chao, S., Chen, X., See, D., & Dubcovsky, J. (2011). Mapping and validation of QTL which confer partial resistance to broadly virulent post-2000 North American races of stripe rust in hexaploid wheat. *Theoretical and Applied Genetics*, *123*(1), 143–157. doi: 10.1007/s00122-011-1573-0
14. Mago, R., Bariana, H. S., Dundas, I. S., Spielmeyer, W., Lawrence, G. J., Pryor, A. J., & Ellis, J. G. (2005). Development of PCR markers for the selection of wheat stem rust resistance genes *Sr24* and *Sr26* in diverse wheat germplasm. *Theoretical and Applied Genetics*, *111*(3), 496–504. doi :10.1007/s00122-005-2039-z
15. Marchal, C., Zhang, J., Zhang, P., Fenwick, P., Steuernagel, B., Adamski, N. M., et al. (2018). BED-domain-containing immune receptors confer diverse resistance spectra to yellow rust. *Nature Plants*, 4(9), 662–668. doi :10.1038/s41477-018-0236-4
16. Mawcha, K. T., Zhang, N., Wang, Y., & Yang, W. (2022). Advances in wheat breeding for resistance to Fusarium head blight. *Czech Journal of Genetics and Plant Breeding*, *58*(4), 167-188. doi :10.17221/1/2022-cjgpb
17. Mukhtar, S., Khan, M. A., Paddar, B. A., Anjum, A., Zaffar, G., Mir, S. A., Kamaluddin. (2015). Molecular characterization of wheat germplasm for stripe rust resistance genes ( *Yr5 , Yr10 , Yr15 & Yr18* ) and identification of candidate lines for stripe rust breeding in Kashmir. *Indian Journal of Biotechnology*.*14*, 241-248
18. Rosewarne, G. M., Singh, R. P., Huerta-Espino, J., William, H. M., Bouchet, S., Cloutier, S., et al. (2005). Leaf tip necrosis, molecular markers and β1-proteasome subunits associated with the slow rusting resistance genes *Lr46/Yr29*. *Theoretical and Applied Genetics*, *112*(3), 500–508. doi :10.1007/s00122-005-0153-6
19. Zhang, M., Ainisai Saimi, Liu, Q., Ma, Z., & Chen, J. (2023). The detection of *Yr* genes in Xinjiang wheat cultivars using different molecular markers. *International Journal of Molecular Sciences*, *24*(17), 13372–13372. doi :10.3390/ijms241713372
